# Multiple myeloma: signaling pathways and targeted therapy

**DOI:** 10.1186/s43556-024-00188-w

**Published:** 2024-07-04

**Authors:** Qizhong Lu, Donghui Yang, Hexian Li, Ting Niu, Aiping Tong

**Affiliations:** 1 Department of Biotherapy, State Key Laboratory of Biotherapy and Cancer Center, Research Unit of Gene and Immunotherapy, Chinese Academy of Medical Sciences, Collaborative Innovation Center of Biotherapy, West China Hospital, Sichuan University, Chengdu, 610041 China; 2 College of Veterinary Medicine, Shaanxi Center of Stem Cells Engineering and Technology, Northwest A&F University, Yangling, 712100 China; 3 Department of Hematology, State Key Laboratory of Biotherapy and Cancer Center, Collaborative Innovation Center of Biotherapy, West China Hospital, Sichuan University, Chengdu, 610041 China; 4 State Key Laboratory of Biotherapy and Cancer Center, Research Unit of Gene and Immunotherapy, Chinese Academy of Medical Sciences, Collaborative Innovation Center of Biotherapy, West China Hospital, Sichuan University, Chengdu, 610041 China; 5 Frontiers Medical Center, Tianfu Jincheng Laboratory, Chengdu, 610212 China

**Keywords:** Multiple myeloma, Signaling pathways, Targeted therapy, Immunotherapies

## Abstract

Multiple myeloma (MM) is the second most common hematological malignancy of plasma cells, characterized by osteolytic bone lesions, anemia, hypercalcemia, renal failure, and the accumulation of malignant plasma cells. The pathogenesis of MM involves the interaction between MM cells and the bone marrow microenvironment through soluble cytokines and cell adhesion molecules, which activate various signaling pathways such as PI3K/AKT/mTOR, RAS/MAPK, JAK/STAT, Wnt/β-catenin, and NF-κB pathways. Aberrant activation of these pathways contributes to the proliferation, survival, migration, and drug resistance of myeloma cells, making them attractive targets for therapeutic intervention. Currently, approved drugs targeting these signaling pathways in MM are limited, with many inhibitors and inducers still in preclinical or clinical research stages. Therapeutic options for MM include non-targeted drugs like alkylating agents, corticosteroids, immunomodulatory drugs, proteasome inhibitors, and histone deacetylase inhibitors. Additionally, targeted drugs such as monoclonal antibodies, chimeric antigen receptor T cells, bispecific T-cell engagers, and bispecific antibodies are being used in MM treatment. Despite significant advancements in MM treatment, the disease remains incurable, emphasizing the need for the development of novel or combined targeted therapies based on emerging theoretical knowledge, technologies, and platforms. In this review, we highlight the key role of signaling pathways in the malignant progression and treatment of MM, exploring advances in targeted therapy and potential treatments to offer further insights for improving MM management and outcomes.

## Introduction

Multiple myeloma (MM) is the second most common hematologic malignancy, accounting for approximately 10% of all hematological cancers, with an annual incidence is approximately 4.5-6 cases per 100,000 individuals. The majority of MM patients are over 40 years old, and approximately 100,000 people worldwide succumb to the disease each year [1–8]. MM typically arises from precursor conditions such as monoclonal gammopathy of undetermined significance (MGUS) and smoldering multiple myeloma (SMM), which originate from abnormal plasma cells in the bone marrow [9]. The hallmark of MM is the uncontrolled proliferation of plasma cells within the bone marrow, leading to the production of excessive and abnormal immunoglobulins [10–13]. The disease is often associated with organ dysfunction that manifests as the “CRAB criteria” in clinical practice, including Calcium elevation (C), Renal dysfunction (R), Anemia (A), and Bone disease (B) [14, 15].

The pathogenesis of MM is complex and involves various factors, including genetic abnormalities [16–22], cytokines production [23–26], suppressive bone marrow microenvironment (BMM) [27–30], and aberrant signaling pathways [23, 31–34]. The interaction between MM cells and the bone marrow microenvironment leads to the activation of intracellular signaling pathways such as PI3K/AKT/mTOR, RAS/MAPK, JAK/STAT, Wnt/β-catenin, and NF-κB pathways. These pathways play crucial roles in promoting the proliferation, migration, expansion, survival, and drug resistance of malignant clones in MM. Targeting proteins that mediate these signaling pathways holds promise as a potential therapeutic approach for MM treatment [32, 35–39]. By disrupting these pathways, it may be possible to inhibit the growth and survival of MM cells, making them more susceptible to conventional therapies such as chemotherapy or targeted agents. Understanding the intricate interplay between MM cells and the bone marrow microenvironment, as well as the signaling pathways involved, is essential for developing novel and effective treatment strategies for MM.

Over the past few decades, several non-targeted drugs have been approved for the treatment of multiple myeloma, including alkylating agents [40], corticosteroids [41–43], immunomodulatory imide drugs (IMiDs) [44–47], proteasome inhibitors (PIs) [42, 48–51], histone deacetylases (iHDACs) inhibitors [37, 52, 53], and XPO1 inhibitor [54–56] (Table 1). While these drugs have significantly extended the survival of patients with multiple myeloma by effectively killing myeloma cells and controlling disease progression, they often come with serious adverse effects due to their non-specificity. The rapid growth of myeloma cells and the challenge of complete resection contribute to the incurable nature of multiple myeloma. Non-targeted drugs, while effective, can also harm normal healthy cells, leading to adverse effects such as gastrointestinal problems (nausea, vomiting, diarrhea, and constipation) [57], myelosuppression (reduction in white blood cells and platelets), alopecia, anemia, liver and kidney impairment, and an increased risk of infections [58, 59]. However, targeted drugs offer a promising avenue for improving treatment efficacy and reducing adverse effects by selectively targeting specific molecules or pathways involved in myeloma cell survival and proliferation. By precisely targeting these key elements, targeted drugs can kill tumor cells more effectively while sparing healthy cells, thus minimizing side effects. Therefore, the development of targeted drugs is crucial for enhancing the treatment outcomes of multiple myeloma patients, offering the potential to achieve better disease control with fewer adverse effects. Continued research and innovation in this area are essential for advancing the field of multiple myeloma treatment and ultimately improving patient outcomes.

**Table 1:** FDA approved non-targeted drugs in the treatment of multiple myeloma

Drugs	Target	Class	Approved time
Melphalan	DNA	Alkylating agent	1960
BiCNU	DNA	Alkylating agent	1982
Cyclophosphamide	DNA	Alkylating agent	1982
Doxorubicin	DNA	Anthracyclin	1982
Thalidomide	Cereblon/E3 ubiquitin ligase complex	IMiD	1998
Bortezomib	Proteasome 20s unit	PI	2003
Lenalidomide	Cereblon/E3 ubiquitin ligase complex	IMiD	2006
Bendamustine	DNA	Alkylating agent	2008
Carfilzomib	Proteasome 20s unit	PI	2012
Pomalidomide	Cereblon/E3 ubiquitin ligase complex	IMiD	2013
Ixazomib	Proteasome 20s unit	PI	2015
Panobinostat	Pan-HDAC	HDCA inhibitor	2015
Selinexor	XPO1	XPO1 inhibitor	2019

In this review, we focus on the key signaling pathways implicated in the pathogenesis of MM and examining the advancements in inhibitors or inducers targeting these pathways for MM treatment. Additionally, the review aims to provide an overview of the progress in targeted therapy and potential therapeutics, including monoclonal antibodies, antibody-drug conjugates, bispecific antibodies, bispecific T-cell engagers, chimeric antigen receptor T (CAR-T) cells, chimeric antigen receptor natural killer (CAR-NK) cells, and T cell receptor-engineered T (TCR-T) cells. Understanding the signaling pathways involved in MM pathogenesis is essential for developing targeted therapies that can effectively disrupt the aberrant cellular processes driving the disease. By targeting specific molecules or pathways within these signaling cascades, inhibitors or inducers can be designed to modulate MM progression and enhance treatment outcomes.

### Signaling pathways of multiple myeloma

**Fig. 1:**
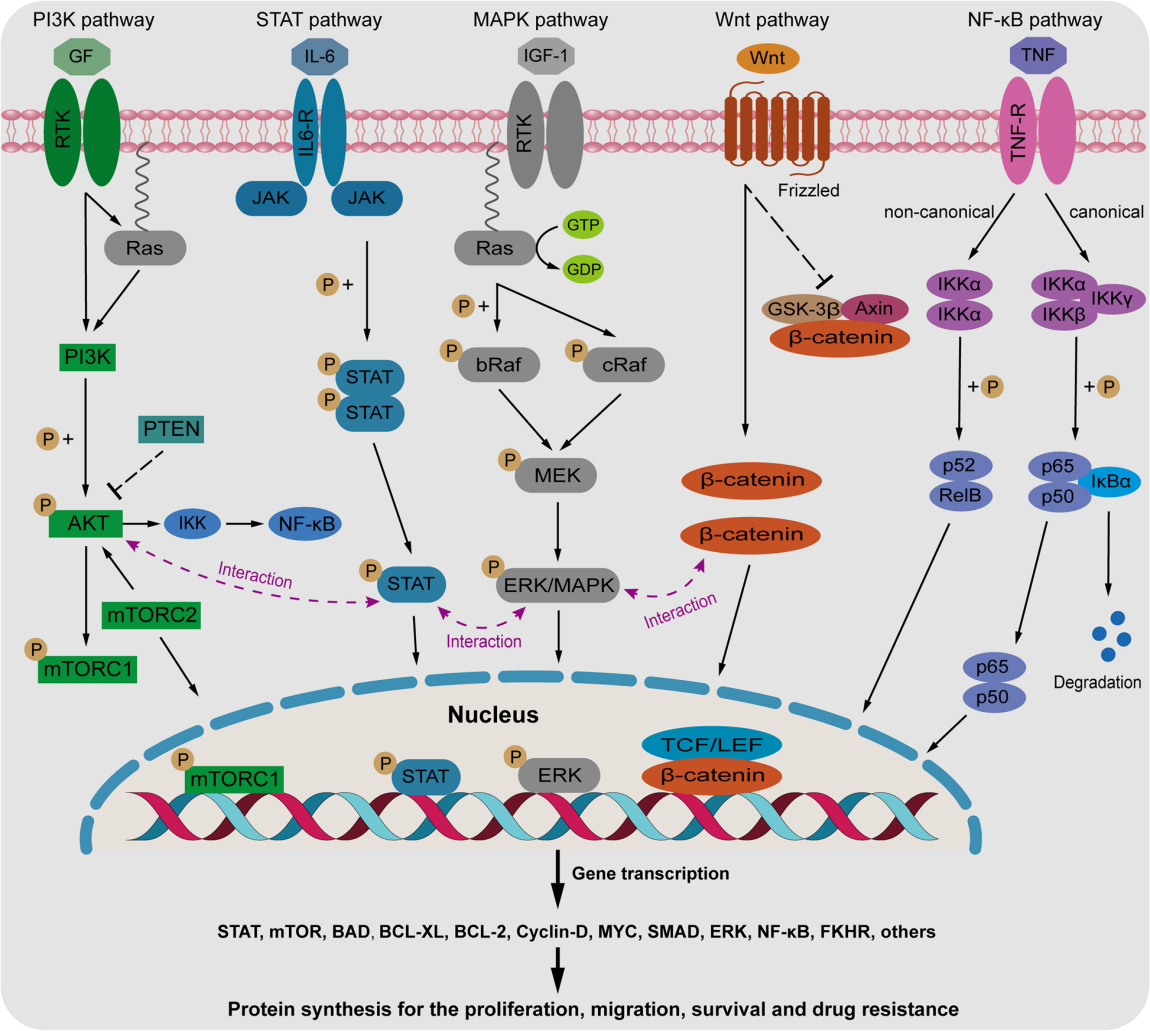
Signaling pathways in the pathogenesis of multiple myeloma. Signaling pathways such as PI3K/AKT/mTOR, JAK/STAT, RAS/MAPK, Wnt/β-catenin and NF-κB pathway participate the pathogenesis of MM by mediating the proliferation, migration, expansion, survival, angiogenesis and drug resistance of MM cells

The pathogenesis of multiple myeloma (MM) involves a complex interplay between genetic abnormalities and the bone marrow microenvironment (BMM), where MM cells interact with various cellular and molecular components. These interactions lead to the activation of multiple intracellular signaling pathways, ultimately promoting the growth, survival, and migration of MM cells. Key signaling pathways implicated in MM pathogenesis include the PI3K/AKT/mTOR, RAS/MAPK, JAK/STAT, Wnt/β-catenin, and NF-κB pathways (Fig. 1). These pathways are dysregulated in MM, driving oncogenic processes and contributing to disease progression [23, 35, 60–64]. Targeting these signaling pathways represent a promising therapeutic strategy for MM. Various inhibitors and inducers have been developed to specifically target components of these pathways, aiming to disrupt oncogenic signaling and inhibit MM cell proliferation and survival.

### PI3K/AKT/mTOR signaling pathway

The PI3K/AKT/mTOR signaling pathway plays a crucial role in MM pathogenesis by regulating various cellular processes such as proliferation, migration, apoptosis, and autophagy. This pathway is frequently activated in MM due to oncogenic events and extrinsic stimulation [65]. In the bone marrow microenvironment, where MM primarily develops, cancerous plasma cells interact closely with non-tumor cells such as stromal and endothelial cells. These interactions result in the increased secretion of interleukin-6 (IL-6) by stromal cells and growth factors by MM cells. IL-6 and growth factors play pivotal roles in MM development and progression. For example, insulin-like growth factor-1 (IGF-1) stimulates the epithelial-mesenchymal transition in MM cells by activating the PI3K/AKT pathway. This transition promotes the formation of migratory MM cells, facilitating tumor growth and dissemination [36]. Moreover, the dissemination of myeloma is associated with the production of IL-6 and tumor necrosis factor-alpha (TNF-α). These cytokines enhance vascular permeability and promote myeloma cell motility by decreasing the adhesion of MM cells to CD138 [66]. As a result, myeloma cells intravasate into the bone marrow, contributing to disease progression.

Several inhibitors targeting the PI3K/AKT/mTOR pathway have been investigated for their anti-tumor effects in both clinical and preclinical studies in the treatment of MM. Temsirolimus, an mTOR inhibitor, has been studied in combination with weekly bortezomib for the treatment of patients with relapsed/refractory MM (RRMM). This combination therapy has shown promising results in clinical trials, indicating its potential efficacy in this patient population [67]. Furthermore, the efficacy of temsirolimus, when combined with the MEK inhibitor trametinib, was found to be enhanced. This combination therapy demonstrated increased anti-tumor activity, suggesting a potential synergistic effect between mTOR and MEK inhibition in MM treatment [68]. Everolimus, another mTOR inhibitor similar to temsirolimus, has been evaluated in combination with bortezomib for its inhibitory effects on MM tumor cells. Studies have shown that this combination therapy has a significant inhibitory effect on MM cells, indicating the potential of everolimus as a treatment option for MM patients [69]. Afuresertib, an oral AKT inhibitor, has been studied as a single agent in MM. It has shown a favorable safety profile in clinical trials, suggesting that AKT inhibition may be a viable therapeutic strategy for MM patients [70]. Zi Y et al. demonstrated that blocking the PI3K/AKT pathway could be achieved by knocking down miR-25-3p. This approach resulted in the inhibition of migration and progression of MM cells [71]. Downregulation of high mobility group protein B2 (HMGB2) by knocking down RP11-301G19.1 was shown to reduce the phosphorylation of PI3K/AKT and significantly inhibit the growth of MM cells [72]. Nitidine Chloride (NC), a small molecule, was found to bind to the target ABCB6, inhibiting the PI3K/AKT pathway and promoting the death of MM cells. This study suggests that ABCB6 may serve as a potential therapeutic target and prognostic biomarker for MM. Additionally, NC emerges as a promising new drug for MM therapy due to its ability to target the PI3K/AKT pathway through ABCB6 inhibition [34]. Thus, inhibition the PI3K/AKT pathway may be a potential therapeutic for the treatment of MM [73].

The cross-talk between signaling pathways plays a crucial role in cellular responses to external stimuli and conditions, allowing cells to adapt and modify their behaviors [74]. Among the well-documented examples of cross-talk, the PI3K/AKT/mTOR and RAS/MAPK pathways exhibit intimate interactions and reciprocal regulation. The experimental findings suggest that PI3K promotes the RAS/MAPK cascade, enhancing ERK responses to physiological stimuli. Activated ERK, in turn, can inhibit the PI3K/AKT pathway [75, 76]. Inhibiting PI3K can boost the activation of RAF, MEK, and/or ERK, while inhibiting MAPK activity leads to decreased PI3K activity [77–79]. In addition, RAS can directly interact and activate PI3K, establishing a direct link between these pathways [80, 81], and the RAS/MAPK pathway also intersects with the PI3K/AKT/mTOR pathway at the mTOR level, further intertwining their signaling [82]. Therefore, the interactions between the PI3K/AKT/mTOR and RAS/MAPK pathways result in complex signaling networks that lead to diverse cellular responses. Simultaneous inhibition of both the PI3K and MAPK pathways has been shown to be more effective in suppressing cancer growth and viability compared to targeting each pathway individually [68, 83].

Overall, the activation of the PI3K/AKT/mTOR pathway in MM cells, coupled with interactions within the bone marrow microenvironment, leads to enhanced cell proliferation, migration, and survival, ultimately promoting tumor growth and dissemination. Targeting this pathway and its interactions with the microenvironment holds promise for developing effective therapeutic strategies against MM.

### RAS/MAPK signaling pathway

The RAS/MAPK pathway plays a crucial role in MM by influencing cell proliferation, survival, and differentiation. The activation of this pathway in MM is often driven by mutations and increased levels of cytokines present in the tumor microenvironment, such as IL-6, insulin-like growth factor (IGF), and vascular endothelial growth factor (VEGF) [84–86]. The aberrant activation of RAS/MAPK pathway contributes to the development and progression of MM and other cancers [87, 88]. The t(4;14) translocation in MM, resulting in the fusion of the fibroblast growth factor receptor 3 gene with an IgH enhancer, leads to overexpression of fibroblast growth factor receptor 3 and subsequent activation of the RAS/MAPK pathway [89]. The mutations of KRAS and NRAS induce the activation of ERK are common in MM, and these mutations present in 23-54% with a new diagnosis, but it increases to 45-81% in RRMM patients [90–92]. It is known that RAS mutations, particularly in KRAS, are associated with a more aggressive phenotype, shorter survival and progression-free survival (PFS), serving as negative prognostic factors in MM [93, 94]. Patients with MM harboring oncogenic KRAS mutations tend to have worse clinical outcomes than those with NRAS mutations or wild-type RAS [95]. The presence of RAS mutations can drive the evolution of MM from an intramedullary disease to a more advanced extramedullary phenotype, further impacting disease progression and prognosis [96–98]. Therefore, the aberrant activation of the RAS/MAPK pathway is closely linked to disease progression in MM and can significantly influence the prognosis of affected individuals. Targeting this pathway and associated mutations may offer promising therapeutic strategies for managing MM and improving patient outcomes.

In the treatment of MM, numerous inhibitors targeting the RAS/MAPK pathway have been developed as therapeutic agents. Among them, AZD4785 stands out as a highly efficient and selective KRAS-targeting antisense oligonucleotide, capable of specifically silencing KRAS and significantly inhibiting MM tumor growth both in vitro and in vivo [99]. AZD4785 has demonstrated good tolerability in preclinical trials and is designed to bind to the 3’UTR of KRAS, targeting all subtypes of KRAS mutations, thus possessing broad therapeutic potential [99]. Additionally, AZD6244 (Selumetinib, ARRY-142886) is a potent ATP non-competitive allosteric MEK1/2 inhibitor that exhibits significant preclinical activity in MM cells [100]. However, a phase 2 study indicated that monotherapy with AZD6244 was well-tolerated in MM patients but showed modest activity [101]. CH5126766/VS-6766 is a novel MEK-pan-RAF inhibitor that has shown durable partial responses and disease stabilization in MM patients harboring KRAS mutations (NCT02407509) [102]. Furthermore, a single-arm, open-label, multicenter phase 2 trial (NCT02834364) evaluated the efficacy and safety of the combination of the BRAF/MEK inhibitors encorafenib and binimetinib in patients with RRMM carrying the BRAFV600E mutation. Results showed an overall response rate (ORR) of 83.3%, a median progression-free survival (PFS) of 5.6 months, and a 24-month overall survival (OS) rate of 55%, indicating significant efficacy of the BRAF/MEK inhibition in patients with BRAFV600E-mutated RRMM [103]. In addition, a phase 2 ROAR trial (NCT02034110) evaluated the efficacy and safety of dabrafenib (BRAF kinase inhibitor) plus trametinib (MEK inhibitor) in MM patients harboring the BRAFV600E mutation, showing an ORR of 50%, a median duration of response (DOR) of 11.1 months, a median PFS of 6.3 months, and a median OS of 33.9 months [104]. Overall clinical findings highlight the significant role of the RAS/MAPK pathway in the treatment of multiple myeloma, with therapeutic strategies targeting this pathway holding crucial implications for improving patient prognosis.

### JAK/STAT signaling pathway

The JAK/STAT pathway was initially discovered in the downstream signaling mediated by interferon-α (IFN-α), IFN-γ, and IL-6. This pathway serves as a crucial signaling cascade that regulates gene expression by transmitting external signals to the nucleus. Activation of the JAK/STAT pathway plays a vital role in controlling the proliferation, differentiation, migration, and apoptosis of tumor cells [39, 63]. Studies have shown that heightened levels of STAT3 expression are linked to a poor prognosis, significantly shorter PFS, and overall survival in MM patients [105, 106]. Furthermore, the abnormal activation of the JAK/STAT pathway has been identified as a key factor driving the development of MM [107]. In MM cells, the JAK/STAT3 pathway is triggered by cytokines from the gp130 receptor family, such as IL-6, leukemia inhibitory factor (LIF), and oncostatin-M (OSM). Within the bone marrow microenvironment, IL-6, an essential cytokine, plays a critical role in the PI3K/AKT/mTOR and RAS/MAPK signaling pathways during the initiation and progression of MM. Additionally, the binding of IL-6 to its receptor (IL-6R) leads to the activation of JAK1, which phosphorylates STAT3, thereby activating the JAK/STAT pathway [108]. Mechanistically, IL-6 induces STAT3-dependent upregulation of MCL1, which enhances the survival of MM cells and confers resistance to the BCL2/BCL-XL inhibitor ABT-737 [109].

Several inhibitors targeting the JAK/STAT pathway have been investigated for their anti-tumor effects in clinical or preclinical studies. Given that IL-6 promotes the proliferation of MM cells, blocking IL-6 is considered a potential strategy for MM treatment [110–112]. Siltuximab, an anti-IL-6 chimeric monoclonal antibody, was the first drug approved for the treatment of multicentric Castleman’s disease (MCD) in the US and EU [113]. In an open-label phase I trial, the combination of Siltuximab with lenalidomide, bortezomib, and dexamethasone (VRD) for newly-diagnosed multiple myeloma (NDMM) showed an overall response rate (ORR) of 90.9%, with 45.5% very good partial response (VGPR) and 36.4% partial response (PR) compared to VRD alone [114]. LLL12, a STAT3 inhibitor, effectively blocked STAT3 phosphorylation, nuclear translocation, DNA binding activity, and induced apoptosis in primary MM cells obtained from patients resistant to lenalidomide and bortezomib [115]. The anti-CD38 monoclonal antibody is commonly used in MM treatment. Studies have revealed that the JAK/STAT3 pathway is involved in the downregulation of CD38, while the JAK/STAT1 pathway is associated with the upregulation of CD38. The JAK inhibitor, ruxolitinib, inhibited STAT3 phosphorylation, increased CD38 expression in MM cells, and enhanced the antibody-dependent cellular cytotoxicity (ADCC) function of the anti-CD38 antibody DARA [116]. A phase I clinical trial (NCT03110822) demonstrated that the JAK inhibitor, ruxolitinib, could overcome resistance to lenalidomide and steroids in patients with RRMM. The clinical benefit rate (CBR) and ORR were 49% and 36%, respectively, with no dose-limiting toxicities reported [117].

The JAK/STAT pathway interacts with the RAS/MAPK and NF-κB signaling pathways, forming a complex network of cross-talk that influences various cellular processes, including cell proliferation, survival, and apoptosis. One example of this interaction is the involvement of c-jun N-terminal kinase (JNK), a member of the MAPK subfamily, in promoting compensatory cell proliferation in tumors. However, the JAK/STAT pathway, through its downstream effector Zfh2, can control the expression of the *fos* gene and the pro-apoptotic *hid* gene, thereby promoting cell survival mediated by JNK and inhibiting JNK-induced cell apoptosis [118]. Furthermore, MAPK has the ability to phosphorylate a serine residue near the C-terminal region of most STAT proteins, indicating a direct interaction between the two signaling pathways [119, 120]. IL-6 plays a pivotal role in connecting the NF-κB and STAT3 signaling pathways. IL-6 and its receptor can activate STAT3, while NF-κB target genes include the encoding of IL-6. Additionally, STAT3 is involved in the activation of the NF-κB pathway, further highlighting the intricate interplay between these pathways [121]. Given the cross-talk of the JAK/STAT, RAS/MAPK, and NF-κB pathways in MM pathogenesis, simultaneous inhibition of these pathways is considered a promising therapeutic approach for MM treatment. Targeting multiple pathways simultaneously may provide more effective and comprehensive treatment strategies for MM therapy.

### Wnt/β-catenin signaling pathway

Wnt signaling is a critical pathway involved in multiple developmental processes and can also contribute to malignant transformation. Wnts comprise a large family of secreted proteins that interact with receptors, consisting of a Frizzled (Fz) family member alone or in complex with LDL receptor-related proteins (LRP5/6), and Wnt signaling mediates multiple developmental processes and can lead to malignant transformation [64]. In the treatment of MM, the bone marrow microenvironment plays a significant role in activating the Wnt/β-catenin pathway through Wnt ligands secreted by bone marrow stromal cells (BMSCs). The aberrant activation of the Wnt/β-catenin pathway is a key feature in the initiation and progression of MM. The dysregulated Wnt signaling in MM has a dual impact, firstly, it promotes the proliferation, migration, and drug resistance of MM cells; secondly, MM cells secrete Wnt antagonists that inhibit osteoblast differentiation, leading to the progression of osteolytic lesions by impeding bone formation [35, 38, 122]. Transcription factors that are aberrantly expressed in MM can regulate the Wnt/β-catenin pathway. For example, the overexpression of KLF10 has been shown to inhibit MM cell activity by reducing the expression of β-catenin, c-Myc, and Cyclin D1, as well as by inhibiting GSK3β phosphorylation [123]. Additionally, research has indicated that the histone demethylase JMJD2C is upregulated in MM tissues, leading to increased transcriptional activity and stability of β-catenin. This upregulation also results in the inhibition of GSK3β protein expression in MM cells, ultimately driving abnormal activation of the Wnt/β-catenin pathway and promoting MM cell proliferation [124].

Non-coding RNAs, transcription factors, proteases, and secreted proteins have been identified as key regulators of the Wnt/β-catenin pathway, exerting significant influence on the development and progression of MM. For example, non-coding RNA, miR-744-5p, inhibits the expression of SOX12, leading to reduced transcriptional activity of β-catenin and TCF/LEF. By suppressing the activity of MM cells, miR-744-5p functions as a negative regulator of the Wnt/β-catenin pathway [125]. In contrast to miR-744-5p, miR-135b activates the Wnt/β-catenin signaling pathway in MM cells. It promotes cell proliferation, migration, and invasion by up-regulating the expression of Wnt-3a, β-catenin, and Cyclin D1, while inhibiting the expression of GSK3β and CK1α [126]. MM cells secrete Wnt inhibitors such as the DKK1 protein, which is crucial for bone homeostasis. Elevated expression of DKK1 induces the phosphorylation of β-catenin in osteoblasts, leading to the inhibition of Wnt/β-catenin pathway activation. This dysregulation contributes to the development of MM osteopathy and accelerates disease progression. Inhibitors targeting DKK1 have shown efficacy in treating MM by restoring Wnt/β-catenin pathway activity [127]. Lenalidomide, a second-generation immunomodulator approved by the FDA for the treatment of MM, has been shown to activate the Wnt/β-catenin pathway in plasma cells, contributing to drug resistance. In detail, lenalidomide treatment leads to the accumulation of β-catenin within plasma cells by inhibiting its degradation and enhancing transcription. The accumulated β-catenin subsequently activates LEF/TCF, transcription factors that regulate gene expression. Activation of LEF/TCF results in the upregulation of target genes such as cyclin D1 and c-Myc, which are associated with cell proliferation and survival [128, 129]. Pyrvinium pamoate (PP), an oral anthelmintic drug, was found to inhibit Wnt signaling by activating CK1α, a member of the destruction complex. This activation led to a reduction in active β-catenin levels. Following PP treatment, both the RPMI-8226 and primary MM cells exhibited an increase in apoptosis, which also reported a synergistic impact on MM cell viability when PP was combined with bortezomib treatment [130].

Signaling cross-talk plays a crucial role in immune regulation, tumorigenesis, and differentiation transcription programs. A study by Ehyai S et al. showed that active MAPK signaling enhances β-catenin nuclear localization and target gene activity. This finding shed light on the mechanistic connection between the RAS/MAPK pathway and the canonical Wnt/β-catenin pathway at a fundamental level [131]. Furthermore, multiple studies have provided evidence of the involvement of MAPK in modulating the activities of the Wnt/β-catenin pathway. These studies collectively highlight the intricate interplay between MAPK signaling and Wnt/β-catenin pathway regulation, emphasizing the importance of signaling cross-talk in cellular processes such as immune response, cancer development, and cellular differentiation [132–135]. The interaction between MAPK and Wnt/β-catenin pathways underscore the complexity of cellular signaling networks and their impact on various physiological and pathological processes. Understanding the cross-talk between these pathways can offer valuable insights into the molecular mechanisms underlying disease states and may pave the way for the development of targeted therapies that exploit these signaling interactions for therapeutic benefit.

Overall, understanding the intricate regulation of the Wnt/β-catenin pathway in MM provides valuable insights into the pathogenesis of the disease and offers potential therapeutic targets for intervention. Targeting components of the Wnt signaling pathway may present opportunities for developing novel treatment strategies to combat MM progression and improve patient outcomes.

### NF-κB signaling pathway

NF-κB is a pivotal transcription factor implicated in MM pathogenesis, particularly in the bone marrow microenvironment. NF-κB is activated by various stimuli, including Toll-like receptor 4 (TLR4) ligands and cytokines such as CD40, TNF-α, IL-1β, and B-cell activating factor (BAFF) within the bone marrow microenvironment. NF-κB signaling is categorized into canonical and non-canonical pathways. In the canonical pathway, NF-κB1 (p50)-RelA (p65) complexes are activated and regulated by a trimeric complex consisting of IκB kinases (IKK) α, β, and γ. Conversely, the non-canonical pathway involves NF-κB2 (p52)-RelB complexes, regulated by a dimeric complex of IKKα [136, 137] (Fig. 1). The aberrant activation of NF-κB signaling is crucial in MM pathogenesis. Approximately 80% of MM patients exhibit elevated levels of NF-κB p65 subunits in bone marrow samples [60, 61, 138, 139]. NF-κB activation regulates the expression of genes controlling proliferation, survival, immortalization, and angiogenesis, including IL-6, IGF-1, and BAFF [140, 141]. Moreover, NF-κB activation mediates bone destruction in MM patients by modulating osteoclast stimulators like MIP-1α [142]. Additionally, it regulates the production of angiogenic factors such as VEGF and angiopoietin-1 (Ang-1), contributing to MM angiogenesis [61, 143]. Importantly, the aberrant activation of the non-canonical NF-κB pathway, along with reduced dependence on the microenvironment, is critical for MM malignant progression [144, 145]. Blocking only the canonical pathway may not sufficiently inhibit NF-κB activity, underscoring the importance of targeting both pathways. In addition, it has demonstrated that the aberrant non-canonical NF-κB signaling induces changes in the epigenome, enhancing transcriptional processes that promote MM progression [146].

Non-canonical NF-κB signaling leads to the overexpression of histone methyltransferase EZH2, which inhibits Myc and H3K27 methylation. This enhances the sensitivity of MM cells to proteasome inhibitors [147]. Bortezomib (BTZ), the first FDA-approved proteasome inhibitor for MM treatment, prolongs overall survival and progression-free survival. It down-regulates IKKα expression and triggers NF-κB activation in MM cells [148]. Despite its efficacy, bortezomib treatment is associated with various side effects, including thrombocytopenia, neutropenia, gastrointestinal toxicity, peripheral neuropathy, infection, and fatigue [48, 149]. While bortezomib has improved MM treatment, a significant proportion of patients do not respond to it, and nearly all patients eventually experience relapse, either with bortezomib alone or in combination therapies [150]. Carfilzomib (CFZ), the second FDA-approved proteasome inhibitor for MM treatment, activates NF-κB, which mediates TNF-α-induced tumor initiation and promotion [151]. PPFIA binding protein 1 (PPFIBP1), highly expressed in MM plasma cells, enhances chemoresistance to bortezomib treatment by stabilizing p65, promoting its cyto-nuclear translocation, and activating the NF-κB signaling pathway [152]. NF-κB inhibitors target tumor cell survival and anti-apoptotic pathways, increasing cell sensitivity to toxic stimuli and low-dose chemotherapy [153–155]. Inhibitors specific to both canonical and non-canonical NF-κB pathways may offer more benefits for MM treatment [156]. Understanding the interplay between NF-κB signaling, proteasome inhibitors, and other molecular pathways is crucial for developing effective therapeutic strategies for MM patients, with the aim of improving treatment response and reducing the risk of relapse and adverse effects.

## Targeted immunotherapy

**Fig. 2:**
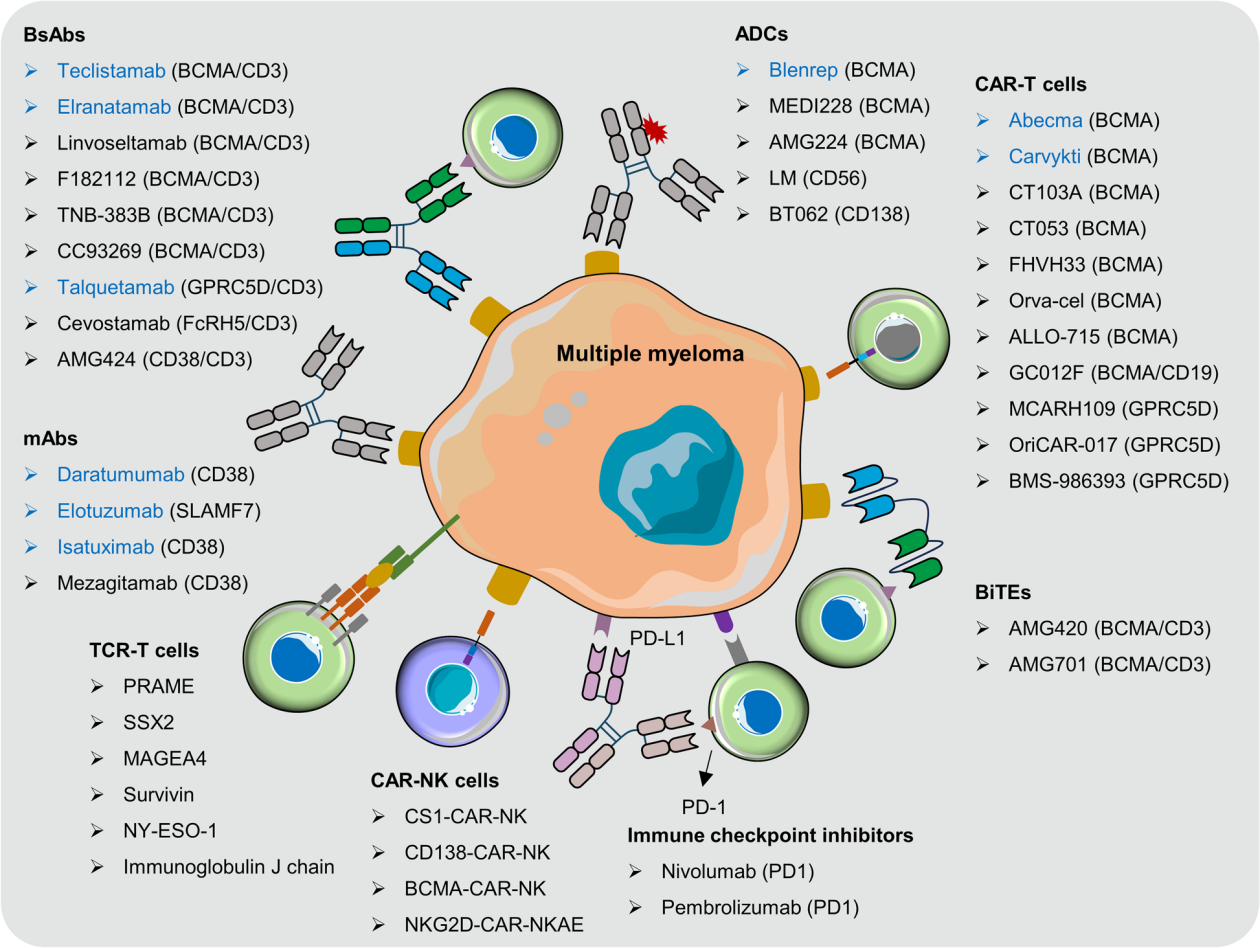
Targeted immunotherapeutic in the treatment of multiple myeloma. Numerous novel targeted immunotherapies have been explored for the treatment of MM, including mAbs, ADCs, BsAbs, ICIs, CAR-T, CAR-NK and TCR-T cells. mAbs: monoclonal antibodies; ADCs: antibody-drug conjugates; BsAbs: bispecific antibodies; BiTEs: bispecific T-cell engagers; ICIs: immune checkpoint inhibitors; CAR-T: chimeric antigen receptor T; BCMA: B cell maturation antigen; GPRC5D: G protein-coupled receptor, class C, group 5, member D; FcRH5: Fc receptor-homolog 5; SLAMF7: SLAM Family Member 7; NKG2D: Natural-killer group 2, member D; PD-1: programmed cell death protein 1; PD-L1: programmed death-ligand 1

Multiple myeloma (MM) is indeed a complex and heterogeneous disease characterized by the dysregulation of multiple signaling pathways that promote its progression, growth, and survival. While there are limited approved drugs targeting these specific pathways in MM, the understanding of targeted genetic alterations in MM has led to the development of specific therapeutic agents tailored to different genetic abnormalities in the disease. In recent years, the field of MM treatment has seen significant advancements in targeted immunotherapies. These novel approaches include monoclonal antibodies (mAbs), antibody-drug conjugates (ADCs), bispecific antibodies (BsAbs), immune checkpoint inhibitors, chimeric antigen receptor T cells (CAR-T), CAR-NK, bispecific T-cell engagers (BiTEs) and TCR-T cells (Fig. 2). These novel immunotherapies offer exciting new possibilities for the treatment of MM, providing additional options for patients who may not respond to traditional therapies or experience relapse. The development and optimization of these targeted immunotherapies hold great promise for improving outcomes in MM patients.

### Monoclonal antibody

The FDA has approved three monoclonal antibodies, daratumumab (anti-CD38), elotuzumab (anti-SLAMF7), and isatuximab (anti-CD38) for the treatment of MM. The use of monoclonal antibodies in the treatment of MM represents a significant advancement, offering targeted and effective therapies that can help control disease progression, improve response rates, and prolong patient survival. These antibodies, either used alone or in combination with other agents, have provided new treatment options for MM patients, particularly those who may have relapsed or become refractory to standard therapies.

#### Daratumumab

Daratumumab is an IgG1 monoclonal antibody that targeting CD38 and induces cell death through complement-dependent cytotoxicity (CDC), antibody-dependent cellular cytotoxicity (ADCC), antibody-dependent cellular phagocytosis (ADCP), and regulation of CD38 enzyme activity [157, 158]. In the CASTOR study, daratumumab in combination with bortezomib and dexamethasone (DVd) demonstrated superior outcomes compared to bortezomib plus dexamethasone (Vd) in patients with RRMM. The overall response rate (ORR) was significantly higher with DVd (85%) compared to Vd (63%). The PFS was also significantly longer with DVd (16.7 months) compared to Vd (7.1 months) [159]. In the phase 3 MAIA trial, daratumumab in combination with lenalidomide and dexamethasone (D-Rd) showed improved PFS compared to lenalidomide plus dexamethasone (Rd) in newly diagnosed multiple myeloma (NDMM) patients who were not eligible for stem cell transplantation. The PFS was 56.2 months with D-Rd versus 34.4 months with Rd [160]. Additionally, the phase 3 trial of daratumumab, bortezomib, lenalidomide, and dexamethasone for MM therapy revealed that the percentage of patients with a complete response or better was higher in the D-VRd group than in the VRd group (87.9% vs. 70.1%, *P* < 0.001), which indicated the addition of daratumumab to VRd induction and consolidation therapy and to lenalidomide maintenance therapy conferred a significant benefit [161]. In the POLLUX phase 3 trial, the median OS was 67.6 months for D-Rd compared with 51.8 months for Rd, D-Rd significantly extended OS compared to Rd alone in patients with RRMM [162]. These findings collectively suggest that daratumumab, whether used as monotherapy or in combination with other therapies, has shown promising efficacy in improving response rates, prolonging progression-free survival, and extending overall survival in patients with multiple myeloma. The combination of daratumumab with other therapeutics has demonstrated enhanced efficacy compared to daratumumab alone, highlighting the importance of combination therapies in the treatment of MM.

#### Elotuzumab

Signaling lymphocyte activation molecule F7 (SLAMF7), also known as CS1/CD319, is a cell surface glycoprotein that plays a crucial role in the immune system. SLAMF7 is highly expressed on multiple myeloma cells. Importantly, SLAMF7 is not found on normal tissues, making it an attractive target for immunotherapy in the treatment of multiple myeloma. Elotuzumab, an IgG1 monoclonal antibody targeting SLAMF7 glycoprotein, is approved for the treatment of RRMM patients in combination with lenalidomide and dexamethasone. In the ELOQUENT-1 study, the efficacy of elotuzumab in combination with lenalidomide and dexamethasone (ERd) was compared to lenalidomide plus dexamethasone (Rd) in NDMM patients who did not undergo autologous stem cell transplantation (ASCT). The study showed that there was no significant difference in PFS between the ERd group (31.4 months) and the Rd group (29.5 months), indicating that elotuzumab did not significantly improve patient survival in this specific group of NDMM patients [163, 164]. Additionally, in the ELOQUENT-2 study, elotuzumab in combination with lenalidomide and dexamethasone (ERd) demonstrated a median PFS of 18.4 months and overall survival of 34 months in patients with RRMM. A high percentage (76.7%) of patients achieved at least a partial remission with ERd therapy, indicating that ERd is a safe and effective regimen for RRMM patients [165]. The ELOQUENT-3 study evaluated the efficacy of elotuzumab in combination with pomalidomide and dexamethasone (EPd) in RRMM patients who had received at least two prior lines of therapy. The study showed that the median overall survival (OS) with EPd was significantly better than with Pd (29.8 months vs. 17.4 months), suggesting that elotuzumab can significantly improve the survival of RRMM patients treated with pomalidomide and dexamethasone [164]. Together, the findings from these studies suggest that elotuzumab, when used in combination with lenalidomide and dexamethasone or pomalidomide and dexamethasone, has the potential to be an effective treatment option for patients with RRMM.

#### Isatuximab

Isatuximab is an FDA-approved humanized monoclonal antibody that targets CD38, a protein highly expressed on MM cells. Isatuximab exerts its anti-tumor effects through various mechanisms, including ADCC, ADCP, and CDC [166]. Isatuximab has been shown to directly induce apoptosis in MM cells without the need for crosslinking agents or effector cells. This unique ability contributes to its effectiveness in killing MM cells [167, 168]. Isatuximab effectively inhibits CD38 enzyme activity compared to other anti-CD38 monoclonal antibodies like daratumumab. By reducing adenosine levels, isatuximab helps alleviate the immunosuppressive environment in the bone marrow, which can support the anti-tumor immune response [167, 169]. In a phase 2 clinical trial, isatuximab monotherapy demonstrated overall response rates (ORRs) ranging from 4.3% to 29.2% in different dose groups. Higher doses of isatuximab (greater than 10 mg/kg) showed improved median progression-free survival (mPFS) and overall survival (OS), with an overall response rate of 40.9% [170]. Based on the positive results from clinical trials, the FDA approved isatuximab in combination with carfilzomib and dexamethasone (Isa-Kd) on March 31, 2021, for the treatment of adult patients with RRMM who had received 1 to 3 prior lines of therapy [171]. In the randomized phase 3 IKEMA trial, isatuximab combined with carfilzomib and dexamethasone significantly reduced the risk of disease progression compared to carfilzomib and dexamethasone alone. The addition of isatuximab to the regimen prolonged median progression-free survival (35.7 vs. 19.2 months) and improved complete response rates and minimal residual disease negativity rates [172]. Overall, isatuximab has shown potent biological activity and controllable efficacy in the treatment of patients with RRMM, providing a valuable addition to the therapeutic options available for this challenging disease.

### Immune checkpoint inhibitors

The use of antibodies targeting immune checkpoint molecules, such as CTLA-4, PD-1, and PD-L1, have shown success in various tumors, leading to FDA approvals for multiple therapeutics. However, the efficacy of these antibodies particularly as monotherapy was limited in the treatment of RRMM [173–176]. A phase 1b clinical data demonstrated limited efficacy of nivolumab and pembrolizumab as single agents in RRMM patients [177]. Additionally, pembrolizumab monotherapy showed limited efficacy in both RRMM and ASCT patients [178]. Although single-agent activity was limited, combining pembrolizumab with lenalidomide or pomalidomide and dexamethasone showed efficacy in single-arm trials. However, these combinations were associated with severe hematological and infectious toxicities, as well as immune-related adverse events such as pneumonia, hypothyroidism, adrenal insufficiency, hepatitis, and vitiligo. These adverse events led to the termination of some clinical trials [179–182]. In addition, other clinical trials investigating PD-1 or PD-L1 inhibitors plus immunomodulators and dexamethasone were also terminated due to serious adverse events [183, 184]. Targeting other immune checkpoint molecules such as LAG3, alone or in combination with XBP1/CD138/CS1 multi-peptide vaccination, holds promise for overcoming immunosuppression and enhancing anti-tumor immune responses in MM. These approaches represent potential avenues for further exploration and development [185]. Together, while immune checkpoint inhibitors have shown promise in other cancers, the efficacy of them particularly as monotherapy in multiple myeloma was limited. The development of combination therapies and exploration of alternative targets are essential for improving outcomes in MM treatment.

### Antibody-drug conjugates

**Fig. 3:**
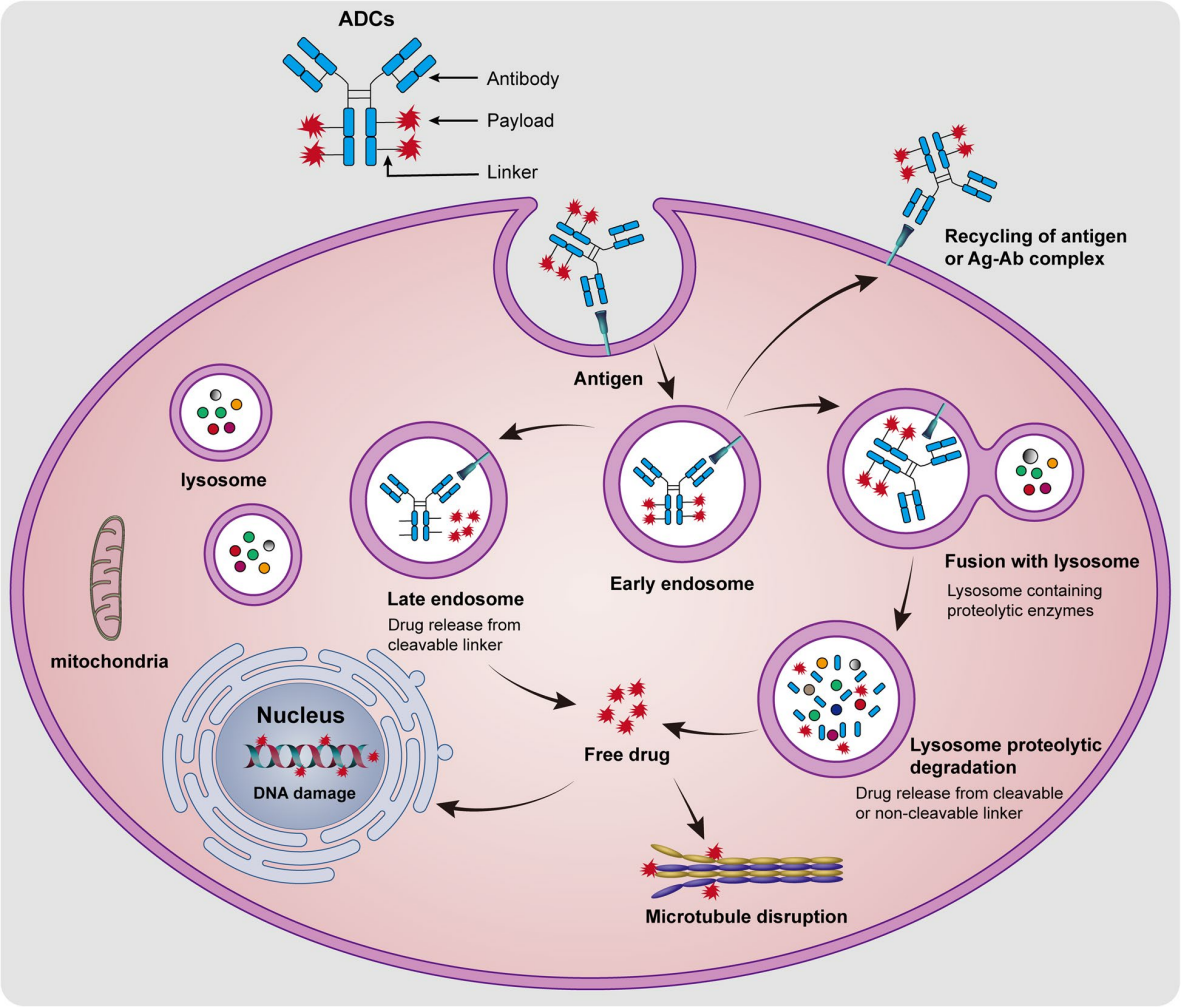
The mechanism of action underlying antibody-drug conjugates in the treatment of multiple myeloma. Circulated ADCs bind to MM cells surface antigens such as BCMA, CD138, CD38, FcRH5 and CD46 through monoclonal antibodies moiety. Bound ADCs are internalized into MM cells in the endosome. The ADC drugs are recycled out of MM cells or released from cleavable linker or digestion via fusion with lysosome. Released drugs induced cell apoptosis or death targeting microtubules or DNA

Antibody-drug conjugates (ADCs) represent a promising class of targeted therapeutics in tumor treatment, combining the specificity of monoclonal antibodies with the cytotoxic effects of chemotherapy. The mechanism of ADCs is shown in the Fig. 3. Once the ADC binds to the tumor-specific antigens, it is internalized by the cancer cell through endocytosis. The ADC-antigen complex is then enclosed within an early endosome. The early endosome matures into late endosomes, which eventually fuse with lysosomes. Within the lysosomes, the cytotoxic payloads are released via chemical or enzyme-mediated mechanisms, ultimately leading to cell apoptosis or death via targeting DNA or microtubule. In some cases, the released cytotoxic payloads may have the ability to diffuse across membranes or affect neighboring cells, even if they did not directly bind to the ADC. This phenomenon is known as the bystander effect and can enhance the overall anti-tumor efficacy of the ADC by affecting a broader range of cancer cells.

#### BCMA-targeted ADCs

Blenrep (belantamab mafodotin-blmf) is a groundbreaking ADC developed by GSK for the treatment of RRMM. It is the first ADC approved by the FDA and EMA for this indication [186]. Blenrep consists of an anti-BCMA antibody called belantamab, linked to the microtubule inhibitor MMAF (monomethyl auristatin F) [186]. In a study involving 18 MM patients, the combination of Blenrep with bortezomib and dexamethasone showed an overall response rate (ORR) of 78%. However, all patients experienced keratopathy as an adverse event, ranging from grade 1 to 3 [187]. In the DREAMM-2 clinical trial (NCT03525678) with 223 RRMM patients who had received three or more prior therapies, Blenrep demonstrated an ORR of 32% and 35% with median PFS of 2.8 and 3.9 months and median overall survival (OS) of 15.3 and 14.0 months for doses of 2.5 mg/kg and 3.4 mg/kg, respectively. The main adverse reactions observed were keratopathy, thrombocytopenia, and anemia [188]. The DREAMM-7 head-to-head phase 3 trial showed positive results for Blenrep, meeting its primary endpoint of PFS. Blenrep combined with bortezomib and dexamethasone significantly extended the time to disease progression or death compared to daratumumab plus bortezomib and dexamethasone [189]. Blenrep, either as a single-agent or in combination with other agents, has demonstrated long-lasting and clinically significant responses in RRMM patients. The safety profile of Blenrep has been considered favorable, with no new safety signals observed in clinical trials. Common adverse reactions include keratopathy, thrombocytopenia, and anemia. The positive results from clinical trials highlight the potential of Blenrep in improving outcomes for patients with RRMM.

In April 2021, AstraZeneca made the decision to discontinue the phase 2 clinical trial of MEDI228, its BCMA-ADC drug, due to the occurrence of severe adverse events among patients with visual impairments [190]. Similarly, Amgen also halted the clinical trials of AMG224, another BCMA-ADC, during its phase 1 trial. Despite these setbacks, there are still ongoing research efforts focused on BCMA-ADC therapy. Among these, notable candidates include HDP-101 and CC-99712. These drugs represent potential alternatives in the pursuit of effective treatments for multiple myeloma [191, 192]. However, it is essential to acknowledge that the development of BCMA-ADC therapies continues to face significant challenges, particularly in addressing adverse events and ensuring patient safety. The discontinuation of clinical trial underscore the complexity of developing targeted therapies for diseases like multiple myeloma, highlighting the need for thorough research and vigilant monitoring of potential side effects.

#### CD138-targeted ADCs

BT062 (Indatuximab ravtansine) is an ADC drug targets CD138. It is created by linking indatuximab to the maytansinoid DM4. Indatuximab binds to CD138 on the surface of MM cells and is internalized. Once inside the cell, the DM4 payload is released into the cytoplasm, where it inhibits microtubule synthesis, leading to cell death [193]. When used as a standalone treatment for patients with RRMM, BT062 has demonstrated effective control of tumor cell growth and has significantly extended patient survival [194, 195]. In a phase 1/2a clinical trial evaluating the safety, efficacy, and pharmacokinetics of BT062 in combination with immunomodulators in RRMM patients who had received at least two prior lines of therapy, promising results were observed. The combination of BT062 with lenalidomide or pomalidomide resulted in objective response rates of 71.7% (33 out of 46 patients) and 70.6% (12 out of 16 patients), respectively. The most common grade 3 to 4 adverse events reported were neutropenia (22%), anemia (16%), and thrombocytopenia (11%) [196]. Recently, another CD138-directed monoclonal antibody, VIS832, has shown stronger binding affinity with CD138 and significant killing activity against both MM cell lines and cells derived from patients with RRMM. VIS832 has also demonstrated efficacy in killing tumor cells that are resistant to daratumumab, another monoclonal antibody that targets CD38. In xenograft models, VIS832 used alone or in combination with bortezomib exhibited potent antitumor activity [197].

#### Other-targeted ADCs

Indeed, besides BT062 and VIS832, there are several other ADCs in development for MM therapy targeting different antigens. TAK-169 and TAK-573 are ADCs targeting CD38, which is highly expressed on the surface of MM cells. These ADCs aim to deliver cytotoxic payloads specifically to CD38-expressing cells, thereby inhibiting tumor growth and inducing cell death [198, 199]. DFRF4539A is an ADC targeting FcRH5, also known as Fc receptor homolog 5. FcRH5 is a cell surface protein that is highly expressed on malignant plasma cells in MM. By directing cytotoxic agents to cells expressing FcRH5, DFRF4539A seeks to eliminate MM cells while sparing normal cells [200]. FOR46 is an ADC targeting CD46, which is a membrane protein involved in regulating complement activation. CD46 is overexpressed in MM, making it a potential target for therapy. FOR46 aims to selectively deliver cytotoxic payloads to CD46-expressing MM cells, leading to their destruction [201, 202]. The development of these ADCs targeting different antigens reflects ongoing efforts to expand the arsenal of treatments available for MM. By leveraging the specificity of antibody targeting and the potency of cytotoxic payloads, these novel therapies hold promise for improving outcomes for patients with MM, particularly those with relapsed or refractory disease.

### Bispecific antibody and BiTEs

**Fig. 4:**
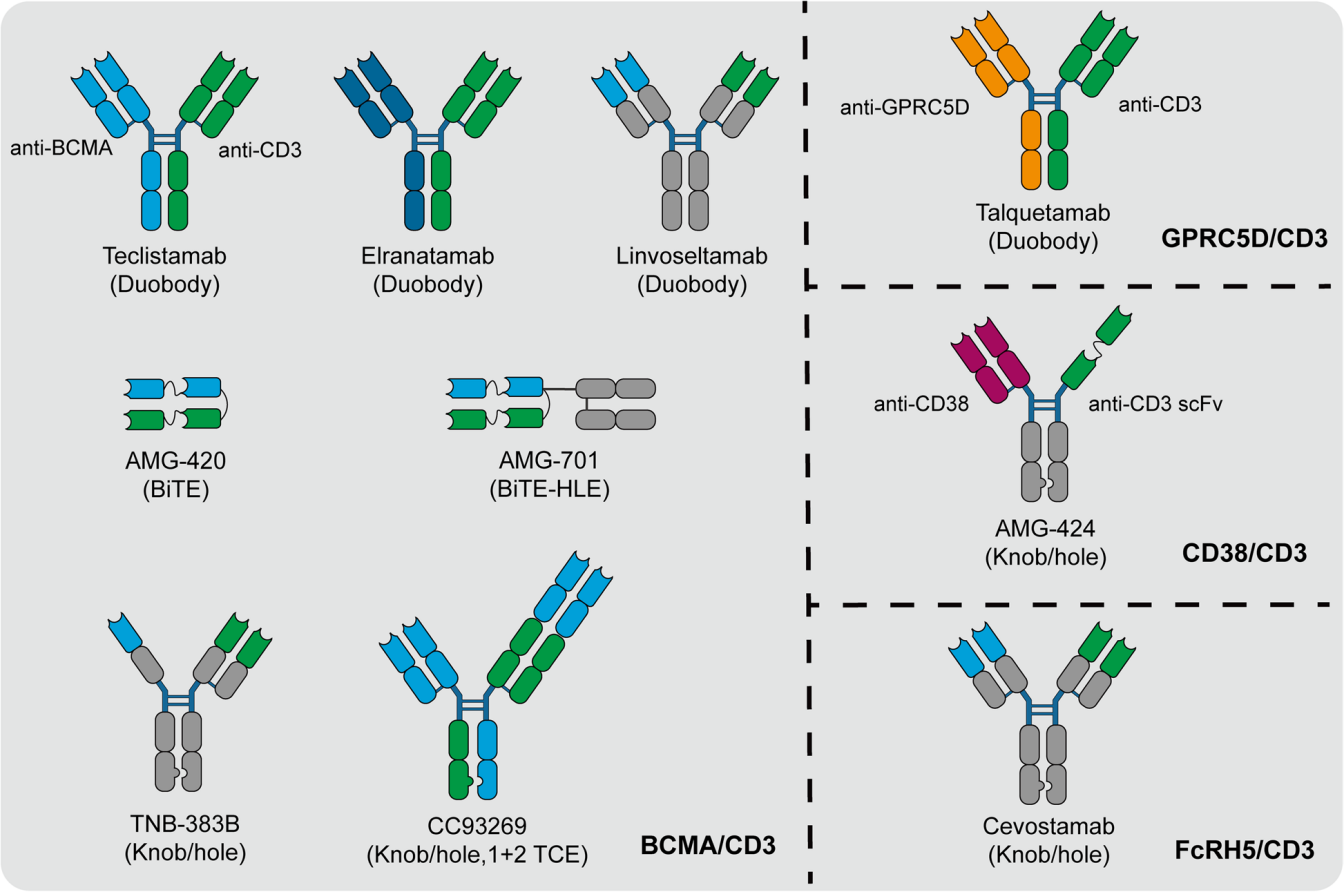
Schematic representation of the T-cell-engaging bispecific antibodies in the treatment of multiple myeloma. T-cell-engaging bispecific antibodies simultaneously bind to antigens on MM cells and CD3 subunits on T cells, leading to the recruitment of T cells to the MM cells, followed by activation, degranulation, and elimination of MM cells. BCMA: B cell maturation antigen; GPRC5D: G protein-coupled receptor, class C, group 5, member D; FcRH5: Fc receptor-homolog 5

Indeed, despite the advancements made with monoclonal antibodies and ADCs in the treatment of MM, overall survival curves have not reached a plateau. To address this, novel targeted immunotherapeutic agents such as T-cell-engaging bispecific antibodies (T-BsAbs) have emerged as promising treatment options. T-BsAbs are designed to simultaneously bind to a tumor-specific epitope and CD3 subunits on T-cells. This dual binding mechanism activates T-cells, leading to their engagement with tumor cells and subsequent killing of the tumor cells. Several T-BsAbs, as well as bispecific T-cell engagers (BiTEs) targeting BCMA, GPRC5D, CD38 and FcRH5 have demonstrated impressive clinical activity in MM patients (Fig. 4). BCMA/CD3-targeted bispecific antibodies, including Teclistamab and Elranatamab, have been approved for the treatment of RRMM in 2022 and 2023, respectively. These agents redirect T-cells to BCMA-expressing MM cells, resulting in tumor cell killing [203, 204]. GPRC5D/CD3-targeted bispecific antibody Talquetamab was also approved for RRMM therapy in 2023. GPRC5D is another antigen highly expressed on MM cells, and Talquetamab facilitates T-cell-mediated killing of MM cells by targeting GPRC5D [205]. Additionally, several other T-BsAbs are currently undergoing clinical trials, for example, Linvoseltamab, F182112, AMG420, AMG701 and Cevostamab [206–208]. The approval and ongoing development of T-BsAbs represent a significant advancement in the field of MM therapy, offering new hope for patients with relapsed or refractory disease.

#### Teclistamab

Teclistamab, a BCMA/CD3-targeted bsAb, has received approval from both the EMA and FDA for the treatment of patients with RRMM who have received at least three prior lines of therapy in 2022 [203]. The approval of Teclistamab was based on data from the MajeTEC-1 clinical trial, which was an open-label, multicenter phase 2 study. In this trial, 165 RRMM patients were treated with Teclistamab, and the following key outcomes were observed 63.0% ORR, 58.8% VGPR, and 39.4% CR. These results demonstrate the significant clinical activity of Teclistamab in RRMM patients who have received multiple prior lines of therapy. The high ORR, VGPR rate, and CR rate indicate substantial anti-myeloma activity of Teclistamab. Additionally, the durable responses observed with a median DOR of 18.4 months, along with the improvements in PFS and OS, highlight the potential of Teclistamab as an effective treatment option for this patient population [209]. The approval of Teclistamab represents a significant milestone in the treatment of RRMM and provides new hope for patients who have exhausted multiple treatment options.

#### Elranatamab

Elranatamab (PF-06863135) is the second BCMA/CD3 bsAb that has been approved for the treatment of patients with RRMM who have received at least four prior lines of therapy, including an anti-CD38 antibody, proteasome inhibitors, and immunomodulators [210]. The approval of Elranatamab was based on data from the MagnetisMM-3 clinical trial, which was a phase 2 study. In this trial, RRMM patients received subcutaneous Elranatamab once weekly. After six cycles, persistent responders switched to biweekly dosing. The objective response rate of Elranatamab reach to 61.0% (75 out of 123 patients); with 35.0%≥CR, and 80.0% of patients maintained their response for at least 6 months [211]. The approval of Elranatamab represents another important advancement in the treatment landscape for RRMM, offering a promising therapeutic option for patients who have exhausted multiple lines of therapy.

#### Linvoseltamab

Linvoseltamab (REGN5458) is a fully human BCMA/CD3-targted bsAb that induces targeted T-cell-mediated cytotoxicity. The LINKER-MM1 phase 1/2 clinical trial (NCT03761108) demonstrated that linvoseltamab as a monotherapy resulted in deep and durable responses in RRMM patients [212, 213]. The efficacy and safety data from the LINKER-MM1 trial suggest that linvoseltamab holds promise as a therapeutic option for patients with RRMM. The deep and durable responses observed indicate the potential of linvoseltamab to effectively target BCMA-expressing myeloma cells and engage T-cell-mediated cytotoxicity to induce anti-tumor responses. Furthermore, early intervention with therapies like linvoseltamab could potentially alter the disease course and improve outcomes for patients by delaying or impeding disease progression.

#### F182112

F182112 is a BCMA/CD3 bsAb, designed to direct both myeloma cells and T-cells to engage in T-cell activation and subsequently cause lysis of BCMA-expressing myeloma cells. The phase 1 clinical trial, NTP-F182112-001 (NCT04984434), aimed to assess the safety, tolerability, pharmacokinetics (PK), and preliminary efficacy of F182112 in patients with RRMM. The trial included 22 patients who were treated with F182112 across eight escalating dose levels ranging from 0.01 to 30 µg/kg. The ORR was reported as 45% (9/20), indicating that 9 out of 20 evaluable patients achieved a response to treatment. 30% (6/20) of patients had stable disease, suggesting a potential benefit in stabilizing disease progression. 25% (5/20) of patients experienced disease progression, indicating that a subset of patients did not respond favorably to the treatment. The favorable safety profile suggests that F182112 has the potential to be administered safely to patients with RRMM [208]. The findings from the phase 1 clinical trial of F182112 suggest that it holds promise as an effective antitumor treatment for patients with RRMM. The observed ORR and tolerability profile are encouraging, indicating that F182112 may offer a viable therapeutic option for this patient population.

#### Talquetamab

GPRC5D (G protein-coupled receptor, class C, group 5, member D) is a G-protein-coupled orphan receptor that highly expressed in malignant bone marrow plasma cells from CD138^+^ MM patients. Its expression in normal tissues is primarily limited to the hair follicle region. Importantly, GPRC5D is expressed independently of BCMA, making it a promising target for multiple myeloma therapy [214, 215]. Talquetamab is the first bispecific antibody that targets GPRC5D/CD3, designed to redirect T-cells to recognize and eliminate GPRC5D-expressing MM cells. Clinical studies have demonstrated the percentages of RRMM patients who responded to talquetamab were 70% (95% CI, 51 to 85) and 64% (95% CI, 48 to 78) at doses of 800 µg and 405 µg per kilogram weekly, respectively. The median duration of response (mDOR) for patients treated with talquetamab at 800 µg and 405 µg per kilogram weekly was 10.2 months and 7.8 months, respectively [216, 217]. On August 9, 2023, the FDA accelerated approval to talquetamab for the treatment of adult RRMM patients who have undergone at least four or more lines of therapy, including a proteasome inhibitor, an immunomodulator, and an anti-CD38 monoclonal antibody [218]. The approval of talquetamab represents a significant advancement in the treatment landscape for RRMM patients who have exhausted multiple lines of therapy. The high response rates and duration of response observed in clinical trials underscore the potential of talquetamab as a valuable therapeutic option for this patient population. Ongoing studies will likely further elucidate the long-term efficacy and safety profile of talquetamab in MM treatment.

#### AMG424

AMG424 is a bsAb targets both CD38 and CD3, designed to redirect T-cells to eliminate MM cells expressing CD38. AMG424 has demonstrated strong anti-tumor activity in vitro against MM cell lines with both low and high expression levels of CD38. This indicates its potential to effectively target a broad range of MM cells. In monkey models, AMG424 exhibited acceptable cytotoxicity and did not induce severe cytokine release syndrome (CRS). This favorable safety profile is crucial for the clinical development of the drug and suggests a lower risk of adverse effects in humans [219]. The promising preclinical data on AMG424, including its strong anti-tumor activity and favorable safety profile, support its further clinical development. The data suggest that AMG424 may have significant activity in MM patients, potentially offering a new treatment option for this patient population. In addition to AMG424, other CD38/CD3-targeted bispecific antibodies such as ISB1342, Y150, and Bi38-3 are also in preclinical development [220–222]. These bsAbs hold promise as future therapeutic options for MM treatment, with the potential to enhance the targeting and elimination of MM cells through CD38-redirected T-cell cytotoxicity. Further preclinical and clinical studies will be necessary to fully evaluate the efficacy and safety of these novel agents in the treatment of MM.

#### Cevostamab

Cevostamab (RG6160) is a bsAb that targets FcRH5 and CD3, designed to engage T-cells for the elimination of MM cells expressing FcRH5. The phase 1 clinical trial (GO39775) indicated that the ORR of RRMM patients who received cevostamab was 58.1% (95% CI, 39.1-77.1), indicating that a significant proportion of patients exhibited a positive response to the treatment. The sCR rates were 6.5% and 6.9% for patients with younger and older than 65 years, respectively, suggesting that some patients achieved a deep and durable response to cevostamab. VGPR rates were 25.8% and 20.7% for patients with younger and older than 65 years, respectively, indicating a substantial proportion of patients had a significant reduction in tumor burden. The common adverse events of CRS were reported in 83.9% and 82.8% of patients, indicating a manageable side effect associated with cevostamab treatment. Grade 3 infection rates were 19.4% and 20.7%, while grade 4 infection rates were 9.7% and 0%, highlighting the importance of monitoring and managing infections during treatment with cevostamab [223]. Based on the positive results from the phase 1 trial and the demonstrated efficacy and tolerability of cevostamab in RRMM patients, the FDA and EMA granted orphan drug designation for cevostamab for the treatment of MM in 2021. This recognition underscores the potential of cevostamab as a valuable therapeutic option for MM patients. Continued research and clinical development will be important to further establish the long-term efficacy and safety profile of cevostamab in the treatment of MM.

#### AMG420

AMG420 (BI 836909), a bispecific T-cell engager (BiTE) targeting BCMA and CD3, represents a novel approach in the treatment of MM. The first-in-human dose-escalation study (NCT02514239) reported an ORR of 70% (7/10) at the dose of 400 µg/d, with 50% of patients achieving minimal residual disease (MRD)-negative complete responses (CRs). The overall response rate across all doses was 31% (13/42), indicating a notable proportion of patients showed a positive response to treatment with AMG420. The adverse events (AEs) associated with AMG420 in the clinical trial were considered acceptable. CRS occurred in 38% of patients, with one patient experiencing a grade 3 event. This is a common side effect of T-cell engaging therapies and can be managed with appropriate supportive care. Infection was reported in 19% of patients, highlighting the importance of monitoring and managing infectious complications during treatment. Peripheral polyneuropathy, a condition affecting the nerves outside of the brain and spinal cord, was observed in 5% of patients as a severe adverse event [224, 225]. A phase 1b multicenter, open-label, expansion study (NCT03836053) is currently underway to further evaluate the safety and efficacy of AMG420 as monotherapy in subjects with RRMM. This study aims to build upon the early clinical data and gather more information on the potential of AMG420 in treating MM. Taken together, the early clinical data from the first-in-human study of AMG420 in patients with RRMM demonstrated promising efficacy, with a significant proportion of patients achieving responses, including MRD-negative complete responses. The safety profile of AMG420 was acceptable, with manageable adverse events such as CRS, infection, and peripheral polyneuropathy. Ongoing studies will provide further insights into the efficacy and safety of AMG420 as a potential treatment option for MM patients, highlighting the continued advancement of innovative therapies in the field of MM treatment.

#### AMG701

AMG701, a half-life extended BiTE targeting BCMA and CD3, showed promising results in the first-in-human phase 1 trial (NCT03287908) for patients with RRMM. The response rate varied across different dose levels. At doses ranging from 3 to 12 mg, the response rate was 36% (16/45), while at a dose of 9 mg, the response rate was notably higher at 83% (5/6). Responses included sCRs, VGPRs, and PRs, indicating a spectrum of positive responses to treatment with AMG701. The most common hematological adverse events included anemia (43%), neutropenia (23%), and thrombocytopenia (20%), highlighting the impact on blood cell counts as a common side effect of treatment. And the most common non-hematological adverse events included CRS (61%), diarrhea (31%), fatigue (25%), and fever (25%). Serious adverse events occurred in 39% of patients and included infections (18%), CRS (9.3%), and asymptomatic pancreatic enzyme rise (3%) [226]. Unfortunately, despite the promising results, the clinical trial of AMG701 monotherapy or in combination with pomalidomide, with or without dexamethasone, was terminated in 2022 due to a business decision. This decision may have been influenced by various factors such as strategic priorities, resource allocation, or changes in the competitive landscape.

### Chimeric antigen receptor T cells

CAR-T cell therapy has revolutionized the treatment of B cell malignancies, including MM, and has shown remarkable efficacy in patients with RRMM. Two BCMA-directed CAR-T products, Abecma and Carvykti, have been approved by the FDA for the treatment of RRMM patients who underwent four or more prior lines of therapy in 2021 and 2022, respectively [227, 228]. Several CAR-T cells targeting antigens, such an GPRC5D [229–231], CD19 [232, 233], CD38 [234–236], CD138 [237], CS1 [238–240] and NKG2D [241, 242] are also under exploration for their efficacy in MM treatment. These antigens represent different targets on MM cells, and targeting them with CAR-T cells may offer additional treatment options for patients with MM (Table 2). Taken together, the approval of BCMA-directed CAR-T therapies like Abecma and Carvykti has marked a significant advancement in the treatment landscape for RRMM. Additionally, the ongoing research and development of CAR-T cells targeting other MM antigens hold promise for further improving outcomes for patients with MM, particularly those with refractory disease or who have relapsed after multiple lines of therapy.

**Table 2:** Clinical trials of CAR-T cells in the treatment of multiple myeloma

Agents	Targets	Class	Antigen binding domain	Dose	ORR and CRR	CRS ≥grade 3	Median follow-up	Median PFS	Median DOR	MRD- negative	Clinical trial ID	Phase	Reference
Abecma	BCMA	Autologus	Mouse scFv	150 × 10^6^ to 450 × 10^6^	ORR: 73%; CRR: 33%	5.0%	13.3 months	8.8 months	12.5 months	26.0%	NCT03361748; NCT04855136; NCT05393804; NCT06045806	Approval	[243, 244]
Carvykti	BCMA	Autologus	Tandem biepitopic VHH	0.75 × 10^6^/kg	ORR: 97%; CRR: 67%	4.0%	12.4 months	Not reached	Not reached	93.0%	NCT03548207; NCT05346835; NCT05347485; NCT04181827	Approval	[245, 246]
CT103A	BCMA	Autologus	Fully human scFv	1.0 × 10^6^/kg	ORR: 96%; CRR: 74.3%	2.8%	13.8 months	Not reached	Not reached	82.4%	NCT05066646; NCT05698303; NCT05181501; NCT05201118; ChiCTR1800018137; ChiCTR2000033946	Phase 1b/2	[247]
FHVH33-CD8BBZ	BCMA	Autologus	Fully human heavy-chain variable domain (FHVH)	4 × 10^6^/kg to 6 × 10^6^/kg	ORR: 96%; CRR: 52%	25.0%	Not reached	78 weeks	18.75 months	Not reached	Not reported	Phase 1	[248, 249]
CT053	BCMA	Autologus	Fully human scFv	0.5 × 10^8^ to 1.8 × 10^8^	ORR: 87.5%; CRR: 79.2%	0.0%	17.4 months	18.8 months	21.8 months	29.1%	NCT03302403; NCT03380039; NCT03716856	Phase 1	[250]
Orva-cel	BCMA	Autologus	Fully human scFv	300 × 10^6^ to 600 × 10^6^	ORR: 91%; CRR: 39%	2.0%	5.9 months	Not reached	Not reached	Not reached	NCT03430011	Phase 1/2	[251]
ALLO-715	BCMA	Allogeneic	Fully human scFv	320 × 10^6^	ORR: 70.8%; CRR: 25%	2.3%	10.2 months	Not reached	8.3 months	93.0%	NCT04093596	Phase 1	[252]
GC012F	BCMA/ CD19	Autologus	Humanized Anti-BCMA scFv and Anti-CD19 scFv	1 × 10^5^/kg to 3 × 10^5^/kg	ORR: 93.1%; CRR: 82.8%	6.9%	30.7 months	38 months	37.0 months	82.8%	NCT04236011; NCT04182581; NCT06235229; NCT05850234; ChiCTR20232951	Phase 1	[253]
BM38 CAR-T	BCMA/ CD38	Autologus	Anti-BCMA scFv and Anti-CD38 scFv	0.5 × 10^6^/kg to 4 × 10^6^/kg	ORR: 87%; CRR: 52%	22.0%	9.0 months	17.2 months	Not reached	87.0%	ChiCTR1800018143	Phase 1	[234]
MCARH109	GPRC5D	Autologus	Mouse scFv	25 × 10^6^ to 150 × 10^6^	ORR: 71%; CRR: 35%	8.3%	10.1 months	16.4 months	7.8 months	100.0%	NCT04555551	Phase 1	[229, 254]
OriCAR-017	GPRC5D	Autologus	Tandem biepitopic VHH	1 × 10^6^/kg to 6 × 10^6^/kg	ORR: 100%; CRR: 60%	0.0%	238 days	Not reached	Not reached	100.0%	NCT05016778; NCT06182696; ChiCTR20232814	Phase 1	[231]
BMS-986393	GPRC5D	Autologus	Mouse scFv	25 × 10^6^ to 150 × 10^6^	ORR: 89.7%; CRR: 47.4%	5.0%	5.9 months	Not reached	Not reached	Not reached	NCT04674813; NCT06121843	Phase 1	[255, 256]
Anti-GPRC5D CAR-T	GPRC5D	Autologus	Unknown	2 × 10^6^	ORR: 91%; CRR: 47.4%	0.0%	5.2 months	Not reached	Not reached	79.0%	ChiCTR2100048888	Phase 2	[257]

*ORR* objective response rate, *CRR* complete response rate, *CRS* cytokine release syndrome, *PFS* progression-free survival, *DOR* duration of response, *MRD* minimal residual disease

### BCMA-targeted CAR-T

#### Abecma

Abecma (idecabtagene vicleucel, ide-cel, bb2121) is a groundbreaking CAR-T cell therapy designed for patients with RRMM who have received at least four prior lines of therapy, including proteasome inhibitors, immunomodulators, and an anti-CD38 monoclonal antibody. Abecma consists of an anti-BCMA single-chain variable fragment (scFv) for targeting BCMA on MM cells. It also contains a 4-1BB co-stimulatory domain to enhance T cell activation and persistence. The CAR-T construct includes a CD3ζ activation domain for T cell activation and killing of MM cells. In the KarMMa study involving 128 patients with RRMM, the overall response rate (ORR) to a single infusion of Abecma was 73%, with a complete response (CR) rate of 33%. The median duration of response (mDOR) was 11 months in patients achieving CR and 19 months in those achieving stringent complete response (sCR). Common side effects such as CRS and immune effector cell-associated neurotoxicity syndrome (ICANS) were mostly low grade, indicating manageable toxicity. Abecma has demonstrated the ability to induce rapid, deep, and long-lasting remissions with just one infusion, providing a new and effective personalized therapy option for MM patients [227, 243, 258, 259]. The approval of Abecma in Europe and Japan further expands the availability of this innovative CAR-T therapy for RRMM patients globally. Overall, Abecma represents a significant advancement in the treatment of RRMM, offering a personalized immunotherapy approach that has shown promising clinical outcomes in terms of response rates, duration of response, and manageable side effects.

#### Carvykti

Carvykti (ciltacabtagene autoleucel, Cilta-cel) is another innovative CAR-T cell therapy developed by Legend Biotech, specifically designed for adult patients with RRMM who have undergone four or more prior lines of therapy [228]. Carvykti is a BCMA-directed genetically modified autologous T cell therapy that utilizes two single-domain antibodies targeting both epitopes of the BCMA antigen. The recommended dose range of Carvykti is 0.5 to 1.0 × 10^6/kg CAR-T cells based on body weight, with patients in the CARTITUDE-1 study receiving a dose of 0.75 × 10^6/kg CAR-T cells. In the phase 1b/2 CARTITUDE-1 study, 94 out of 97 patients treated with Carvykti experienced early, deep, and long-lasting responses. The overall response rate (ORR) was an impressive 97% (95% CI, 91.2-99.4), with 67% of patients achieving stringent complete response (sCR). At the 12-month follow-up, all patients who received Carvykti maintained early, sustained, and deep remissions, highlighting the durable efficacy of the treatment. Additionally, at a median 28-month follow-up, patients treated with Carvykti continued to show deep and long-lasting responses, demonstrating the sustained effectiveness of the therapy in RRMM patients. The most common hematological adverse events associated with Carvykti treatment included neutropenia (95%), anemia (68%), leukopenia (61%), thrombocytopenia (60%), and lymphocytopenia (50%). CRS occurred in 95% of patients, with 4% of cases classified as grade 3 or 4. ICANS occurred in 21% of patients, with 9% of cases being grade 3 or 4 [246]. Carvykti has shown remarkable efficacy in treating RRMM patients, with high response rates, durable remissions, and a manageable safety profile. The approval of Carvykti by the FDA provides another valuable treatment option for patients with advanced multiple myeloma.

#### CT103A

CT103A is a fully human BCMA-directed CAR-T cell therapy that has shown promising results in the treatment of RRMM. CT103A was granted Regenerative Medicine Advanced Therapy (RMAT) and Fast Track Designation (FTD) by the FDA for the treatment of RRMM on February 11, 2023. National Medical Products Administration (NMPA) approved CT103A for the treatment of adult RRMM patients after at least 3 prior lines of therapy on June 30, 2023. In the phase 1b/2 study (FUMANBA-1) involving 103 patients with RRMM who received CT103A (17 in phase 1b; 86 in phase 2) with a median follow-up of 13.8 months. The median time to the first response was 16 days (range 11-179), and a remarkable 96% ORR was observed, with 74.3% achieving at least a CR. The median duration of response (DOR) and median PFS had not reached at the longer median follow-up of 13.8 months [247]. CT103A represents a promising BCMA-directed CAR-T cell therapy for the treatment of relapsed or refractory multiple myeloma, with robust clinical efficacy and the potential to address the unmet medical needs of patients with advanced disease.

#### FHVH33-CD8BBZ

FHVH33-CD8BBZ is a fully human anti-BCMA CAR-T cell therapy with a heavy-chain-only antigen-recognition domain, 4-1BB co-stimulatory domain, and CD3ζ activation domain. FHVH33-CD8BBZ CAR-T cells were administered to 25 patients with relapsed multiple myeloma at a dose of 4-6 × 10^6/kg CAR-T cells. Patients had received a median of 6 prior lines of therapy before receiving FHVH33-CD8BBZ infusions. Results indicated that 92% (23/25) patients achieved an objective response, 68% (17/25) of patients attained sCR or VGPR, and the overall PFS was 78 weeks, indicating a durable response to treatment. 25% of patients experienced a maximum CRS grade of 3, and no patients had CRS greater than grade 3 [248, 249]. FHVH33-CD8BBZ CAR-T cell therapy represents a promising treatment approach for patients with relapsed multiple myeloma, offering high response rates, durable responses, and a favorable safety profile. The therapeutic efficacy in patients with low BCMA expression further underscores its potential to address the unmet medical needs in this patient population.

#### CT053

CT053 is an autologous anti-BCMA CAR-T cell therapy that expresses a fully-human BCMA-specific scFv, a 4-1BB co-stimulatory domain, and a CD3ζ activation domain. In the phase I studies (NCT03302403, NCT03380039, NCT03716856), 24 patients received a dose ranging from 0.5 to 1.8 × 10^6 CT053 cells, the ORR was 87.5%, indicating a high rate of response to CT053 treatment, with 79.2% patients achieved CR (12.5%) or sCR (66.7%), demonstrating deep and durable responses, and the median follow-up time was 17.4 months, the mPFS was 18.8 months and the mDOR was 21.8 months. Anti-drug antibody (ADA) was not detected in patients after infusion, suggesting no significant immunogenicity associated with CT053 treatment, and CT053 demonstrated strong efficacy and a good safety profile for patients with RRMM [250].

#### Orva-cel

Orva-cel (JCARH125) is a BCMA-targeted CAR-T cell therapy that utilizes a fully human scFv, an optimized spacer, and incorporates the 4-1BB costimulatory domain and CD3ζ activation domain. In the phase 1/2 EVOLVE study (NCT03430011), patients with RRMM received orva-cel at 300, 450, and 600 × 10^6 CAR-T cells. Prior to receiving orva-cel, patients underwent lymphodepletion with fludarabine/cyclophosphamide to optimize the efficacy of the CAR-T cell therapy. The ORR was 91% and 39% of patients achieved a CR/sCR. After a median follow-up of 5.9 months, the median PFS was not reached, suggesting prolonged disease control and favorable outcomes in terms of disease progression. Grade ≥ 3 infections occurred in 14% of patients, indicating that some patients experienced severe infections as a side effect of treatment [251]. Based on the results of the EVOLVE study, Orva-cel demonstrated high efficacy in patients with RRMM. The therapy achieved a high overall response rate, with a substantial proportion of patients achieving deep responses such as CR or sCR. Additionally, the therapy showed promising results in terms of PFS, with a mPFS that was not reached at the time of the analysis. However, it is important to note that some patients experienced grade ≥ 3 infections, highlighting the importance of monitoring and managing potential adverse events associated with CAR-T cell therapy.

#### GC012F

GC012F is an autologous CAR-T cell therapy that targets both BCMA and CD19 antigens, known as a dual-targeting FasTCAR-T cell therapy [260]. In an open-label phase I study of GC012F for patients with RRMM. GC012F was administered as a single infusion at doses ranging from 1 × 10^5/kg to 3 × 10^5/kg. Patients underwent a standard 3-day lymphodepletion regimen prior to receiving GC012F. The reported the ORR was 93.1%, sCR was 82.8%, and VGPR was 89.7%. All patients achieved MRD negativity. The median follow-up was 30.7 months, mDOR was 37.0 months, and mPFS was 38.0 months [253]. Additionally, in a single arm, open-label phase I study of GC012F for patients with high-risk (HR) newly-diagnosed multiple myeloma (NDMM). The ORR was 100% and sCR was 95.5%. The median follow-up was 13.6 months, median DOR and PFS were not reached at the time of analysis [261]. Compared to traditional single-target therapies, CD19/BCMA dual-target design of GC012F is expected to enhance treatment efficacy and reduce the probability of antigen escape-induced relapse compared to traditional single-target therapies. However, further information on the safety profile and long-term outcomes of GC012F would be necessary to fully assess its clinical utility and safety profile.

#### BM38 CAR-Ts

BM38 CAR-Ts are humanized bispecific CAR-T cells designed to target both BCMA and CD38 antigens for the treatment of patients with RRMM. The phase I trial evaluating the efficacy and safety of BM38 CAR-T therapy, 87% (20/23) patients achieved a clinical response, and 20 responders achieved MRD-negativity, sCR was 52% (12/23). The median follow-up was 9.0 months (range 0.5 to 18.5), the mPFS was 17.2 months. The CRS occurred in 87% (20/23) of patients and grade ≥ 3 CRS occurred in 22% of patients. Hematologic toxicities were common, including neutropenia (96%), leukopenia (87%), anemia (43%) and thrombocytopenia (61%) [234]. Despite the occurrence of CRS and hematologic toxicities, the therapy was deemed safe and highly effective in this patient population. Further studies and longer follow-up periods may provide additional insights into the durability and long-term outcomes of BM38 CAR-T therapy in RRMM patients.

#### GPRC5D-targeted CAR-T

Although BCMA-directed CAR-T cells therapy has shown high objective response rates in the treatment of RRMM patients, its efficacy is not promising for patients with low or negative BCMA expression, and most patients still experience resistance and progression, which led to the exploration of alternative targets for the treatment of MM. One such promising target is GPRC5D, which has shown independent expression from BCMA and has demonstrated excellent anti-tumor activity in MM models with BCMA loss. Several GPRC5D-targeted agents are currently being evaluated in clinical trials for the treatment of MM (Table 3), and these agents can be developed as both single-targeted therapies focusing solely on GPRC5D or as dual-targeted therapeutics in combination with BCMA-directed therapies [214, 262]. Further research and clinical trials will be crucial in determining the full potential of GPRC5D as a therapeutic target and its role in improving outcomes for MM patients.

**Table 3:** Clinical trials of GPRC5D-targeted agents in the treatment of multiple myeloma

Agent	Target	Class	ClinicalTrials Identifier	Clinical Phase
Talquetamab-tgvs	GPRC5D/CD3	BsAb	NCT04773522	Approval
QLS32015	GPRC5D/CD3	BsAb	NCT05920876	Phase 1
BCMA-GPRC5D CAR-T	BCMA/GPRC5D	CAR-T	NCT05998928	Phase 2
Anti-BCMA/GPRC5D CAR-T	BCMA/GPRC5D	CAR-T	NCT05509530	Phase 2
Anti-BCMA/GPRC5D CAR-T	BCMA/GPRC5D	CAR-T	NCT05509530	Phase 2
OriC321	BCMA/GPRC5D	CAR-T	NCT05325801	Phase 1
Anti-BCMA/GPRC5D CAR-T	BCMA/GPRC5D	CAR-T	NCT05431608	Phase 1
MCARH109	GPRC5G	CAR-T	NCT04555551	Phase 1
Anti-GPRC5D CAR-T	GPRC5G	CAR-T	NCT05749133	Phase 1/2
CAR-GPRC5D	GPRC5G	CAR-T	NCT05759793	Phase 1
Anti-GPRC5D CAR-T	GPRC5G	CAR-T	NCT05739188	Phase 1/2
CAR-GPRC5D	GPRC5G	CAR-T	NCT05219721	Phase 1
OriCAR-017	GPRC5G	CAR-T	NCT05016778	Early Phase 1

#### MCARH109

The phase 1 clinical trial study of MCARH109, a GPRC5D-targeted CAR-T cell therapy, in patients with RRMM has provided valuable insights into the efficacy and safety of this novel treatment approach. A total of 17 patients with RRMM were enrolled in the phase 1 clinical trial of MCARH109, including relapse after BCMA-directed CAR-T cells therapy. Among the patients who received MCARH109 therapy, a response was reported in 71% (12/17) of the entire cohort. Specifically, 58% of patients who received doses ranging from 25 × 10^6 to 150 × 10^6 cells achieved a response. Of the total patients, 35% (6/17) achieved a CR, while 59% (10/17) achieved VGPR or better. The most common grade 3 or higher adverse events (AEs) included neutropenia (94%), thrombocytopenia (65%), and anemia (35%), which confirm that GPRC5D is an active immunotherapeutic target in multiple myeloma [229, 254]. The safety profile of MCARH109, with manageable adverse events, supports further exploration of GPRC5D-targeted therapies in larger clinical trials and potentially in combination with other treatment modalities.

#### OriCAR-017

The POLARIS phase 1 clinical trial is examined the safety and efficacy of OriCAR-017, a GPRC5D-targeted CAR-T cell therapy, in patients with RRMM. The trial was a first-in-human, single-center, single-arm study, enrolling 10 GPRC5D-positive RRMM patients aged from 18 to 75 years who had received at least 3 prior lines of therapy. These patients were intravenously administered 1 × 10^6/kg, 3 × 10^6/kg, and 6 × 10^6/kg of CAR-T cells. During a median follow-up of 238 days, no serious adverse events or treatment-related deaths were reported. The observed adverse events included neutropenia (100%), thrombocytopenia (90%), leukopenia (90%), and anemia (70%). CRS was observed in all patients, with 90% classified as grade 1 and 10% as grade 2. No neurotoxicity was reported. The therapy demonstrated a good response rate, with 60% of patients achieving sCR and 40% achieving VGPR. The favorable response rates suggest that GPRC5D-directed CAR-T cells like OriCAR-017 have potential as a safe and effective therapy for RRMM patients [231]. Overall, the POLARIS study provides initial evidence for the potential of GPRC5D-targeted CAR-T cells as a treatment option for patients with RRMM, warranting further investigation in larger, multicenter trials to confirm these findings and optimize dosing and safety protocols.

#### BMS-986393

BMS-986393 (CC-95266) is a GPRC5D-targeted autologous CAR-T cells therapy in patients with RRMM. The clinical trial involving BMS-986393 in patients with RRMM showed promising results. The study involved dose escalation of BMS-986393 at doses of 25, 75, 150, 300, and 450 × 10^6 CAR-T cells. The ORR across all patients was 86% (55/64), and 75% (21/28) in patients previously treated with BCMA-directed therapies, and the CR was 38% (24/64). In refractory patients to previously BCMA-directed therapies, the ORR was 85% (11/13), CR was 46% (6/13), and median follow-up was 5.9 months. CRS occurred in 85% of patients, with grade ≥ 3 in 4% patients, 4% of patients experienced hemophagocytic lymphohistiocytosis (HLH). In this study, BMS-986393 demonstrated a favorable safety profile and strong, long-lasting responses, including achieving MRD negativity across all dose levels tested, even in patients who did not respond to previous BCMA-directed therapies [255, 256]. The results from the study suggest that BMS-986393 is a promising CAR-T cell therapy for RRMM patients, with high response rates and durable responses.

#### Anti-GRPC5D CAR-T

The phase II, single-arm study of an anti-GPRC5D CAR-T therapy (ChiCTR2100048888) for patients with RRMM has shown promising results. From September 1, 2021, to March 23, 2022, 33 patients with RRMM were enrolled in the study, and received with 2 × 10^6 anti-GPRC5D CAR-T cells. Results showed that the median follow-up was 5.2 months, the ORR was 91% (30/33), 33% (11/33) sCR, 30% (10/33) CR, 12% (4/33) VGPR, 15% (5/33) PR, and 79% (26/33) achieved bone marrow MRD negativity. The therapy showed efficacy even in patients who had progressed after anti-BCMA CAR-T cell therapy, with 100% (9/9) of these patients having partial responses or better. CRS occurred in 76% (25/33) of patients, with all events being grade 1 or 2, indicating manageable toxicity [257].

Based on these results, the anti-GPRC5D CAR-T cell therapy appears to be a potential treatment option for RRMM patients who have progressed after anti-BCMA therapy. The high ORR, particularly for patients with prior anti-BCMA exposure, suggests that targeting GPRC5D could be an effective strategy for a subset of patients resistant to BCMA-directed therapies. However, further research, including larger trials and expanded patient populations, is needed to validate these findings and to better understand the long-term safety and efficacy of the therapy.

### Universal chimeric antigen receptor T cells

The use of autologous CAR-T cells has shown remarkable efficacy in the treatment of RRMM. However, there are several limitations associated with autologous CAR-T cell therapy, including cost, long preparation time, and variability in T cell quality. To address these challenges, the development of universal CAR-T (UCAR-T or allogeneic CAR-T) therapy has emerged as an innovative approach. By utilizing gene-editing techniques to knock out genes such as the T cell receptor (TCR) and other genes responsible for individual immunogenicity, the risk of graft-versus-host disease (GvHD) and host-versus-graft rejection (HvGR) can be minimized. This approach allows for the creation of off-the-shelf CAR-T cell products that can be readily available for patients, reducing the time and cost associated with personalized autologous CAR-T cell therapy [263, 264]. Universal CAR-T cells can be manufactured in advance and stored for immediate use, eliminating the need for the lengthy and personalized production process required for autologous CAR-T cells. The industrialization of UCAR-T therapy can lead to economies of scale, making CAR-T cell therapy more affordable and accessible to a larger patient population. By using healthy donor-derived T cells, the variability in T cell quality observed in autologous CAR-T therapy can be minimized, leading to more consistent treatment outcomes. Despite the potential advantages of UCAR-T therapy, there are still challenges to overcome, such as ensuring the safety and efficacy of allogeneic CAR-T cells and managing potential immune reactions in recipients.

ALLO-715 is a first-in-class, allogeneic, anti-BCMA CAR-T therapy that has shown promising results in the treatment of RRMM. The phase 1 study included 24 RRMM patients who were treated with a dose of 320 × 10^6 ALLO-715 CAR-T cells. Prior to CAR-T cell infusion, patients underwent a lymphocyte clearance regimen consisting of fludarabine, cyclophosphamide, and the anti-CD52 antibody ALLO-647. The ORR was 70.8%, with VGPR in 45.8% of patients and the CR or sCR in 25% of patients. The median DOR was 8.3 months. Adverse events included CRS in 55.8% of patients, neurotoxicity in 14% of patients, and infections in 53.5% of patients. Despite these adverse effects, patients who achieved sustained and effective remission demonstrated the potential benefit of ALLO-715 therapy [252]. BC404-UCART is a BCMA-directed universal CAR-T cell therapy developed by Lu Q et al. The CAR-T cells are engineered to secrete a CD47-SIRPα blocker using the CRISPR/Cas9 gene-editing system. BC404-UCART cells were shown to significantly inhibit the growth of MM tumor cells and prolong the survival of mice in a xenograft model. BC404-UCART cells represent an “off-the-shelf” immunotherapy approach, providing a more readily available and standardized treatment option for MM patients. The use of a CD47-SIRPα blocker may enhance the efficacy of CAR-T cell therapy by overcoming immune evasion mechanisms employed by tumor cells [265]. Therefore, universal CAR-T therapy holds great promise in overcoming the limitations of autologous CAR-T cell therapy and could facilitate the industrialization and wider adoption of CAR-T cell therapy for the treatment of RRMM and other cancers. Continued advancements in gene-editing technologies and clinical research are crucial for realizing the full potential of UCAR-T therapy in improving patient outcomes and expanding access to innovative cellular therapies.

### CAR-NK cells

After CAR-T cell infusion, a phenomenon known as CRS can occur due to the release of a large number of cytokines by the CAR-T cells in response to target cell stimulation. CRS can lead to various non-specific immune responses in patients, resulting in symptoms such as fever, myalgia, low blood pressure, respiratory difficulties, coagulation disorders, and end-organ dysfunction. However, NK cells have a different cytokine secretion profile compared to CAR-T cells. NK cells only secrete several cytokines such as interferon-gamma (IFN-γ), granulocyte-macrophage colony-stimulating factor (GM-CSF), perforin, and granzyme. Importantly, NK cells do not produce IL-1 and IL-6, which are key cytokines involved in the development of CRS [266, 267]. Due to this difference in cytokine secretion profile, NK cells have the potential to be developed as “off-the-shelf” CAR products with a lower risk of inducing CRS compared to CAR-T cells. By leveraging the unique characteristics of NK cells, CAR-NK cells could offer a safer and more controllable approach for adoptive cellular therapy in the future [267–272].

CAR-NK cell therapy has shown promising results in targeting MM by utilizing different CARs to enhance the cytotoxicity of NK cells against MM cells. Some of the targets of CAR-NK cell therapy for MM include BCMA, CS1, CD138, and NKG2D [273–276]. In vitro studies have shown that CS1-CAR-NK cells specifically bind to CS1-positive MM cells, leading to increased lysis of MM cells and enhanced production of IFN-γ. In a xenograft mouse model of MM, CS1-CAR-NK cells effectively suppressed the growth of human MM cells and prolonged mouse survival [273]. Research has demonstrated that CD138-CAR-NK cells enhance the cytotoxic effect of NK cells against MM cells, promote the secretion of granzyme B, IFN-γ, and expression of CD107a. In a NOD-SCID xenograft mouse model, CD138-CAR-NK cells exhibited strong anti-tumor activity against MM [274]. Leivas A et al. developed NKG2D-CAR-NK cells have shown significant killing activity against MM tumor cells in vitro and have effectively controlled MM cell growth in vivo, as demonstrated by preclinical studies [275]. According to Ren Q reported, single-domain antibody (VHH)-directed BCMA CAR-NK cells secreting IL-15 have exhibited remarkable specific killing activity against MM cells. This makes them a promising candidate for immunotherapy in the treatment of MM [276]. Taken together, NK cells and CAR-NK cells have the potential to be developed as “off-the-shelf” products for MM treatment. However, further preclinical and clinical trials are necessary to evaluate and enhance the safety and efficacy of NK cell therapy for MM.

### TCR-T cells

TCR-T cell therapy has shown relative effectiveness in the treatment of solid tumors compared to CAR-T cell therapy due to the ability of TCR-T cells to recognize both intracellular and extracellular proteins. This broadens the range of targets that can be recognized by TCR-T cells, making them potentially more versatile in targeting cancer cells [277]. In a study conducted by Lulla PD et al., T cells were enriched from five myeloma-related targets capable of responding to natural TCR in vitro, including PRAME, SSX2, MAGEA4, Survivin, and NY-ESO-1. Subsequently, 21 patients with RRMM were treated with TCR-T cells, which resulted in well-tolerated treatment and significantly prolonged PFS [278]. Additionally, research by Meeuwsen MH et al. demonstrated that immunoglobulin J chain-targeted TCR-T cells effectively eradicated MM cells in a preclinical in vivo model, leading to a 100-fold lower tumor burden compared to control-treated mice. This highlights the potential of TCR-T cells in targeting multiple myeloma cells and reducing tumor burden [279]. Furthermore, compared to CAR-T cells, TCR-T cells may be more feasible for creating personalized therapies rather than “off-the-shelf” products for RRMM patients. This suggests that MM patients who do not respond to other immunotherapies may benefit from the selection of TCR-T cells for treatment. In conclusion, TCR-T cell therapy shows promise in the treatment of multiple myeloma, particularly in patients with relapsed/refractory disease. Further research and clinical trials are needed to fully evaluate the efficacy and safety of TCR-T cell therapy in the treatment of multiple myeloma.

## Conclusion and perspectives

Multiple myeloma (MM) remains an incurable hematologic malignancy, despite the development of various advances in treatment approaches over the past two decades. In the bone marrow microenvironment, soluble factors such as cytokines and cell adhesion molecules activate intracellular signaling pathways in MM cells, promoting uncontrolled proliferation, survival, migration, apoptosis inhibition, and drug resistance. Therefore, therapeutic agents targeting cytokine-dependent signaling pathways to induce specific death of myeloma cells represent an effective targeted therapeutic strategy for MM. In the pathogenesis of MM, several crucial signaling pathways play important roles. Targeting these pathways through specific therapies can effectively interfere with the survival and proliferation of MM cells, thereby achieving therapeutic efficacy. Some therapies act on cytokine-dependent signaling pathways, inhibiting their activation and inducing apoptosis of multiple myeloma cells or suppressing their growth.

Targeted therapies for multiple myeloma offer significant advantages such as effectiveness in halting disease progression, improving symptoms, and increasing overall survival. These treatments are highly specific, targeting cancer cells while sparing healthy tissue and cells. CAR-T and CAR-NK cells offer innovative alternatives to autologous therapies, potentially leading to more cost-effective and accessible treatment options. The development of targeted therapies has advanced understanding of MM biology, which may lead to better treatment strategies in the future. Nevertheless, targeted therapy still faces several challenges, including high treatment costs that limit access for many patients, suboptimal progression-free survival rates, the risk of relapse due to resistance mechanisms, and potential side effects like CRS and neurotoxicity. Financial constraints may lead to treatment discontinuation, hindering the full potential of these therapies.

Novel therapeutic strategies for MM are rapidly evolving. Enhancing the specificity and efficacy of targeted therapies to further minimize off-target effects and improve treatment outcomes is one of the key current directions. At the same time, the development of more affordable treatment options to increase accessibility to a wider patient population is also a key goal. It is also critical to investigate combination therapies to overcome resistance mechanisms and improve long-term outcomes. In addition, strategies that combine targeted therapies with signaling pathways include targeting multiple pathways simultaneously to enhance therapeutic efficacy and overcome resistance. Personalizing treatment regimens for individual patients is crucial to enhance therapeutic effectiveness. Close monitoring and management of side effects are essential for maintaining treatment adherence and improving patient outcomes. By addressing these key developments and implementing a strategic combination approach, the field of targeted therapies for multiple myeloma can continue to progress and provide patients with improved outcomes and quality of life.

Future advancements in monoclonal antibodies may involve early combination with other antitumor agents such as dexamethasone, IMiDs, PIs, and XPO1 inhibitors. Additionally, the development of new monoclonal antibodies targeting specific antigens is a key focus for further progress. For CAR-T cells, enhancement options include the utilization of new targets or dual-targets like GPRC5D, FcRH5, NKG2D, CD46, BCMA/CD19, BCMA/GPRC5D, BCMA/CS1, and GPRC5D/FcRH5. Refining the preparation process and exploring combination therapies with other antitumor agents are also important strategies to improve CAR-T cell therapy efficacy. Furthermore, combining ADCs, bispecific antibodies, and CAR-T cells with other agents hold promise for enhancing outcomes in early relapse cases. However, the efficacy and optimal combinations of these novel therapies need to be validated through preclinical data and clinical trials. Ongoing research is expected to bring more novel drugs into clinical, significantly improving survival rates and prognosis. This progress may potentially transition the treatment approach for multiple myeloma towards a chronic disease management model or achieve long-term disease control.

## Data Availability

Not applicable.

## Abbreviations

ADA: Anti-drug antibody
ADCC: Antibody-dependent cellular cytotoxicity
ADCP: Antibody-dependent cellular phagocytosis
ADCs: Antibody-drug conjugates
AEs: Adverse events
Allo-SCT: Allogeneic stem cell transplantation
ASCT: Autologous stem cell transplantation
BAFF: B-cell activating factor
BiTEs: Bispecific T-cell engagers
BMM: Bone marrow microenvironment
BMSCs: Bone marrow stromal cells
BsAbs: Bispecific antibodies
CAR: Chimeric antigen receptor
CBR: Clinical benefit rate
CDC: Complement-dependent cytotoxicity
CK1α: Casein kinase 1α
CRS: Cytokine release syndrome
CTLA4: Cytotoxic T lymphocyte-associated antigen-4
DKK1: Dickkopf-1
EMA: European Medicine Agency
FDA: Food and Drug Administration
GPRC5D: G protein-coupled receptor, class C, group 5, member D
GSK3β: Glycogen synthase kinase 3β
GvHD: Graft versus host disease
HDAC: Histone deacetylase
HMGB2: High mobility group protein B2
HvGR: Host versus graft reaction
ICANs: Immune effector cell-associated neurotoxicity syndrome
IGF: Insulin-like growth factor
IKK: IκB kinasse
IL-6: Interleukin-6
IMiDs: Immunomodulatory imide drugs
JMJD2C: Jumonji domain containing protein 2C
KLF10: Krüppel-like factor 10
LIF: Leukemia inhibitory factor
mAbs: Monoclonal antibodies
mDOR: median duration of response
MGUS: Monoclonal gammopathy of undetermined significance
MM: Multiple myeloma
MMAF: Monomethyl auristatin F
mPFS: medain progression-free survival
NC: Nitidine Chloride
NDMM: Newly diagnosed multiple myeloma
NK cells: Natural killer cells
ORR: Overall response rate
OS: Overall survival
OSM: Oncostatin-M
PCs: Plasma cells
PFS: Progression-free survival
PIs: Proteasome inhibitors
PPFIBP1: PPFIA binding protein 1
RRMM: Relapsed/refractory multiple myeloma
sCR: Stringent complete response
SMM: Smoldering multiple myeloma
TCF/LEF: T-cell factor/lymphoid enhancer factor
TCR: T cell receptor
TNF-α: Tumor necrosis factor-α
VEGF: Vascular endothelial growth factor
VGPR: Very good partial response
